# A Non-Coding RNA Promotes Bacterial Persistence and Decreases Virulence by Regulating a Regulator in *Staphylococcus aureus*

**DOI:** 10.1371/journal.ppat.1003979

**Published:** 2014-03-20

**Authors:** Cédric Romilly, Claire Lays, Arnaud Tomasini, Isabelle Caldelari, Yvonne Benito, Philippe Hammann, Thomas Geissmann, Sandrine Boisset, Pascale Romby, François Vandenesch

**Affiliations:** 1 Architecture et Réactivité de l'ARN, Université de Strasbourg, CNRS, IBMC, Strasbourg, France; 2 CIRI, International Center for Infectiology Research, Université de Lyon, Lyon, France; 3 Inserm U1111, Lyon, France; 4 Ecole Normale Supérieure de Lyon, Lyon, France; 5 Université Lyon 1, Lyon, France; 6 CNRS, UMR5308, Lyon, France; 7 Hospices Civils de Lyon, Lyon, France; 8 Plateforme Protéomique IBMC, Strasbourg, France; Harvard Medical School, United States of America

## Abstract

*Staphylococcus aureus* produces a high number of RNAs for which the functions are poorly understood. Several non-coding RNAs carry a C-rich sequence suggesting that they regulate mRNAs at the post-transcriptional level. We demonstrate that the Sigma B-dependent RsaA RNA represses the synthesis of the global transcriptional regulator MgrA by forming an imperfect duplex with the Shine and Dalgarno sequence and a loop-loop interaction within the coding region of the target mRNA. These two recognition sites are required for translation repression. Consequently, RsaA causes enhanced production of biofilm and a decreased synthesis of capsule formation in several strain backgrounds. These phenotypes led to a decreased protection of *S. aureus* against opsonophagocytic killing by polymorphonuclear leukocytes compared to the mutant strains lacking RsaA. Mice animal models showed that RsaA attenuates the severity of acute systemic infections and enhances chronic catheter infection. RsaA takes part in a regulatory network that contributes to the complex interactions of *S. aureus* with the host immune system to moderate invasiveness and favour chronic infections. It is the first example of a conserved small RNA in *S. aureus* functioning as a virulence suppressor of acute infections. Because *S. aureus* is essentially a human commensal, we propose that RsaA has been positively selected through evolution to support commensalism and saprophytic interactions with the host.

## Author Summary

*Staphylococcus aureus* is a commensal and an opportunistic pathogen that causes a large range of community and hospital-acquired infections. The bacteria produce an array of virulence factors, the expression of which is regulated by a set of regulators including proteins and RNAs. In recent years, a large number of small non-coding RNAs encoded by the *S. aureus* genome have been identified but determination of their function is still lagging behind. This study shows that RsaA, a staphylococcal conserved non-coding RNA, operates at the post-transcriptional level by repressing the translation of the master regulatory protein MgrA. The repression is based on a direct interaction of RsaA with the ribosome binding site of *mgr*A mRNA. Through MgrA regulation, RsaA activates biofilm formation and inhibits capsule synthesis. Using appropriate animal models, we showed that RsaA acts as a suppressor of virulence because the deletion of its gene increases the invasiveness of *S. aureus* in the mice sepsis model. RsaA is thus part of complex regulatory network that modify the interactions of *S. aureus* with the eukaryotic immune system. These findings illustrate how small RNAs can have a major impact in bacterial biology.

## Introduction

*Staphylococcus aureus* is an opportunistic pathogen that has evolved complex regulatory circuits allowing rapid adaption of cell growth in response to its diverse hosts and ecological niches. Present in a large proportion of the population as a commensal of skin and nose, the bacteria is also responsible for a large range of hospital-acquired and community infections [1]. A successful infection by *S. aureus* largely depends on the coordinated and sequential expression of a multitude of virulence factors and accessory genes. Over the last decade, it has been established that *S. aureus* genes are regulated at many different levels by a variety of trans-acting regulators, which act in a coordinated manner [2], [3]. Among them, RNAs are now recognized as important players in virulence and many physiological and adaptive responses [4], [5]. The first regulatory RNA that was discovered in 1993 is RNAIII, the main intracellular effector of the quorum sensing *agr* system [6]. This multi-functional regulatory RNA binds to several target mRNAs to regulate their translation and decay [7]–[11]. Later on, several teams have experimentally identified a large number of small RNAs (sRNA) that are issued from the core genome and from mobile and accessory elements (e.g., [12], [13]). These sRNAs include *cis*-acting regulatory regions of mRNAs (the so-called riboswitches), *cis*-encoding antisense RNAs (asRNA), and non-coding RNAs (ncRNAs). In addition, sRNAs carrying small open reading frames (sORF) have been recently identified [14]–[17] and one of them was shown to express a small cytolytic peptide [17]. The functional and mechanistic studies of *trans*-acting sRNAs are still lagging behind their discovery, but some of them are known to sense population density and various environmental changes, modify cell surface properties, adjust the bacterial metabolism during cell growth, regulate virulence gene expression, and respond to antibiotic treatment [4], [18].

We have previously identified a group of sRNAs that carry a conserved and unpaired UCCC sequence suggesting that they act through a common regulatory mechanism [12]–[14], [19]–[23]. This sequence motif is located in several hairpin loops of *S. aureus* RNAIII and was demonstrated to be the seed sequence, which recognizes the ribosome binding sites of mRNA targets to repress translation [5]. Here, using a combination of *in vivo* and *in vitro* approaches, we have elucidated the function and the mechanism of action of one of these sRNAs called RsaA [20]. We show that RsaA, a Sigma B (σ^B^)-dependent sRNA, represses the translation of *mgr*A mRNA, which codes for a master regulator of transcription [24]–[28]. Through MgrA regulation, RsaA activates biofilm formation and inhibits capsule synthesis. Experimental animal models showed that RsaA attenuates the severity of acute systemic infections and enhances chronic catheter infection. These data indicate that RsaA is part of a regulatory circuit that contributes to the complex interactions of *S. aureus* with the eukaryotic immune system.

## Results

### RsaA affects the synthesis of MgrA and of proteins regulated by MgrA

**Figure 1:**
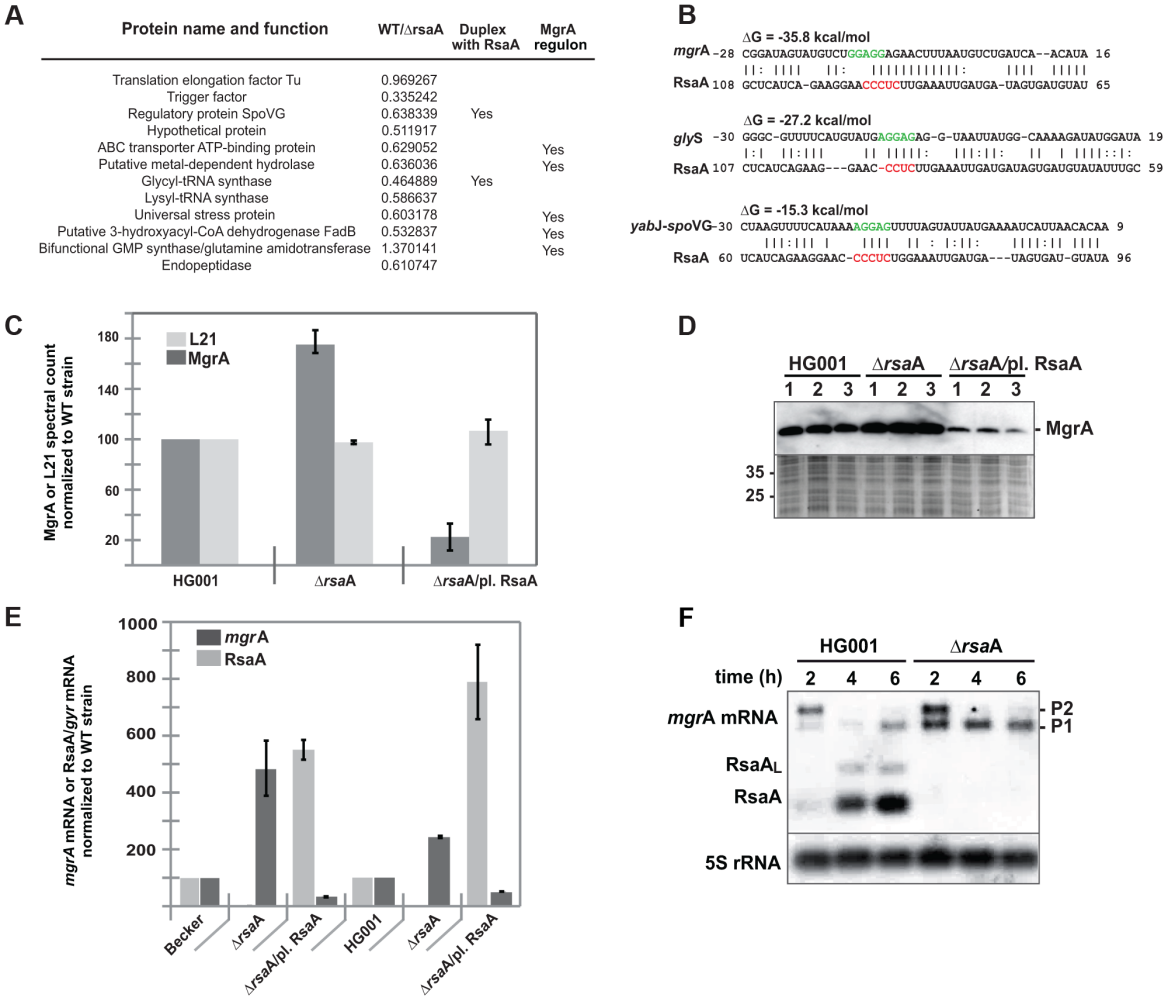
RsaA regulates the synthesis of several metabolic enzymes. (A) 2-D fluorescence difference gel electrophoresis (DiGE) performed on HG001 and Δ*rsa*A-HG001 strains reveals proteins whose synthesis is affected by the deletion of RsaA. The proteins were identified by mass spectroscopy analysis. Ratios correspond to the quantification obtained for HG001 versus Δ*rsa*A strain (data from four independent experiments). In the last column are given the proteins, which belong to the MgrA regulon as shown by Luong et al. [26]. Statistical differences (p<0.05, except for the bifunctional GMP synthase : p<0.01) between the two strains were obtained for each protein. (B) Potential base pairings between RsaA and potential mRNA targets are shown. The Shine and Dalgarno sequence (SD) of target mRNAs are shown in green while the conserved C rich motif in RsaA is in red. Other predicted RsaA-mRNA complexes are shown in Table S1. (C) Relative MgrA protein expression level was defined by LC-MS-MS followed by a spectral counting strategy. Protein extracts were prepared from HG001 (WT), Δ*rsa*A mutant strain, and the same strain complemented with a plasmid expressing RsaA. In parallel to MgrA, we analyzed ribosomal protein L21 as internal control of constitutively expressed protein. The spectral count was normalized to the value determined for the WT strain. (D) Western blot performed with monoclonal antibodies against MgrA performed on protein extracts prepared from HG001, Δ*rsa*A mutant strain and the same strain complemented with a plasmid expressing RsaA. As a control, a gel run in parallel with the same samples was stained with Coomassie blue to verify that each lane contained comparable amounts of protein. Protein size markers were run in parallel. (E) The expression of RsaA and of *mgr*A mRNA was quantified and normalized to the level of *gyr*B mRNA expression from total RNA extracts prepared from *in vitro* culture to the mid- or late-exponential phase of HG001 (WT), the Δ*rsa*A mutant strain, and the same strain complemented with a plasmid expressing RsaA. (F) Northern experiments showing the steady state levels of *mgr*A mRNA in HG001 and in the Δ*rsa*A mutant strains. Total RNAs were prepared after 2, 4 and 6 h of culture in BHI at 37°C. We used two different RNA probes to visualize the expression of RsaA and of *mgr*A mRNA. We have previously shown that a longer transcript including RsaA was also expressed [20]. This longer form of RsaA is known to be processed by RNase Y [37].

To assess the function of RsaA, we first constructed an HG001-derived strain, in which the *rsa*A gene was deleted and replaced by *aph*A-3 (aminoglycoside 3′-phosphotransferase, kanamycin resistance). We have verified that RsaA expression in HG001, which is σ^B^-dependent, was maximal at the late-exponential phase of growth and was abolished in the mutant Δ*rsa*A strain. In BHI medium, growth was not affected by *rsa*A deletion. We then analyzed the effect of the Δ*rsa*A mutation on protein synthesis using comparative proteomics performed on cytosolic protein extracts prepared from wild-type (HG001) and mutant Δ*rsa*A strains grown in BHI medium to late-exponential growth phase. Protein samples were pre-labeled with two different fluorescent dyes (Cy3 and Cy5) and were separated by a two-dimensional difference gel electrophoresis (DiGE). Even though only a small proportion of cytosolic proteins (around 100 proteins) were analyzed, we identified more than 10 proteins with significantly altered expression patterns (Figures 1A, S1). Among the proteins whose synthesis was significantly increased in the mutant strain, was SpoVG, a protein involved in capsule synthesis [29]. Other proteins involved in intermediary metabolism and in virulence were also affected by the Δ*rsa*A mutation. Several of these proteins belong to the regulon of the pleiotropic regulator MgrA [25], [26] but the effect of RsaA was opposite to that of MgrA.

RsaA contains an unpaired and conserved C-rich sequence, suitable for interaction with ribosome binding sites (RBS) of mRNAs, as previously shown for several other regulatory RNAs in *S. aureus* [12]–[14], [19]–[23]. Using IntaRNA [30] and targetRNA [31], we searched for potential intermolecular base pairings between the ribosome binding sites (RBS) of mRNAs (30 nucleotides (nts) downstream and 20 nts upstream of the AUG codon) and the conserved unpaired region of RsaA. Interestingly, the best mRNA candidate obtained by the two programs was *mgr*A mRNA (Table S1). In addition, two mRNAs encoding SpoVG and glycyl-tRNA synthetase, whose protein levels were decreased in an RsaA-dependent fashion, were predicted to form base pairings with RsaA (Figure 1B).

Because MgrA protein could not be detected by the differential proteomic analysis, two alternative methods were used to analyze whether the synthesis of MgrA was under the control of RsaA. Protein extracts, prepared from the WT strain (HG001), the Δ*rsa*A-HG001 mutant strain, and the same strain complemented with a plasmid expressing RsaA under its own promoter were analyzed by Western blot using monoclonal antibodies (generously provided by A. Cheung) and by LC-MS-MS coupled to spectral count analysis (Figure 1C, D). The data obtained by the two methods were well correlated and showed that the yield of MgrA protein was decreased by two-fold in the WT strain as compared to the Δ*rsa*A mutant strain while the repression was even more pronounced (about 10-fold) in the mutant strain expressing RsaA from a plasmid. This effect was specific since the yield of the ribosomal protein L21 remained identical in the various strains (Figure 1C).

We have previously shown that *S. aureus* RNAIII, which represses translation of target mRNAs, also affects their turnover. Therefore, we analyzed the steady-state level of *mgr*A mRNA using quantitative RT-PCR analysis on total RNA extracts prepared from the WT (HG001 and Becker) strains, the Δ*rsa*A mutant strains, and the same mutant strains transformed with a plasmid expressing RsaA (Figure 1E). The data showed that the level of *mgr*A mRNA was strongly enhanced in the Δ*rsa*A mutant strains while complementation caused an even greater decrease in mRNA yield than in the isogenic WT strains (Figure 1E). Because *mgr*A is transcribed in two transcripts [27], which initiate at −124 or −302 nts upstream of the initiation codon, northern experiments were also performed (Figure 1F). The level of the longest mRNA (from P2 promoter) decreased significantly at the late exponential phase of growth in both the WT and Δ*rsa*A derivative of HG001, indicating that the growth phase-dependent regulation of the P2 promoter is not under the control of RsaA (Figure 1F). Conversely, the level of the second and smaller transcript (from P1 promoter) was strongly enhanced in the mutant strain lacking the *rsa*A gene (Δ*rsa*A). These data suggested that the *mgr*A mRNA transcribed from the P1 promoter is under the control of RsaA while the mRNA formed from the P2 promoter is regulated independently of RsaA.

All in all, these data advocated that RsaA has a significant effect on the *S. aureus* proteome most probably through indirect interactions via the regulation of *mgr*A.

### RsaA binds to *mgr*A mRNA at two distinct regions

**Figure 2:**
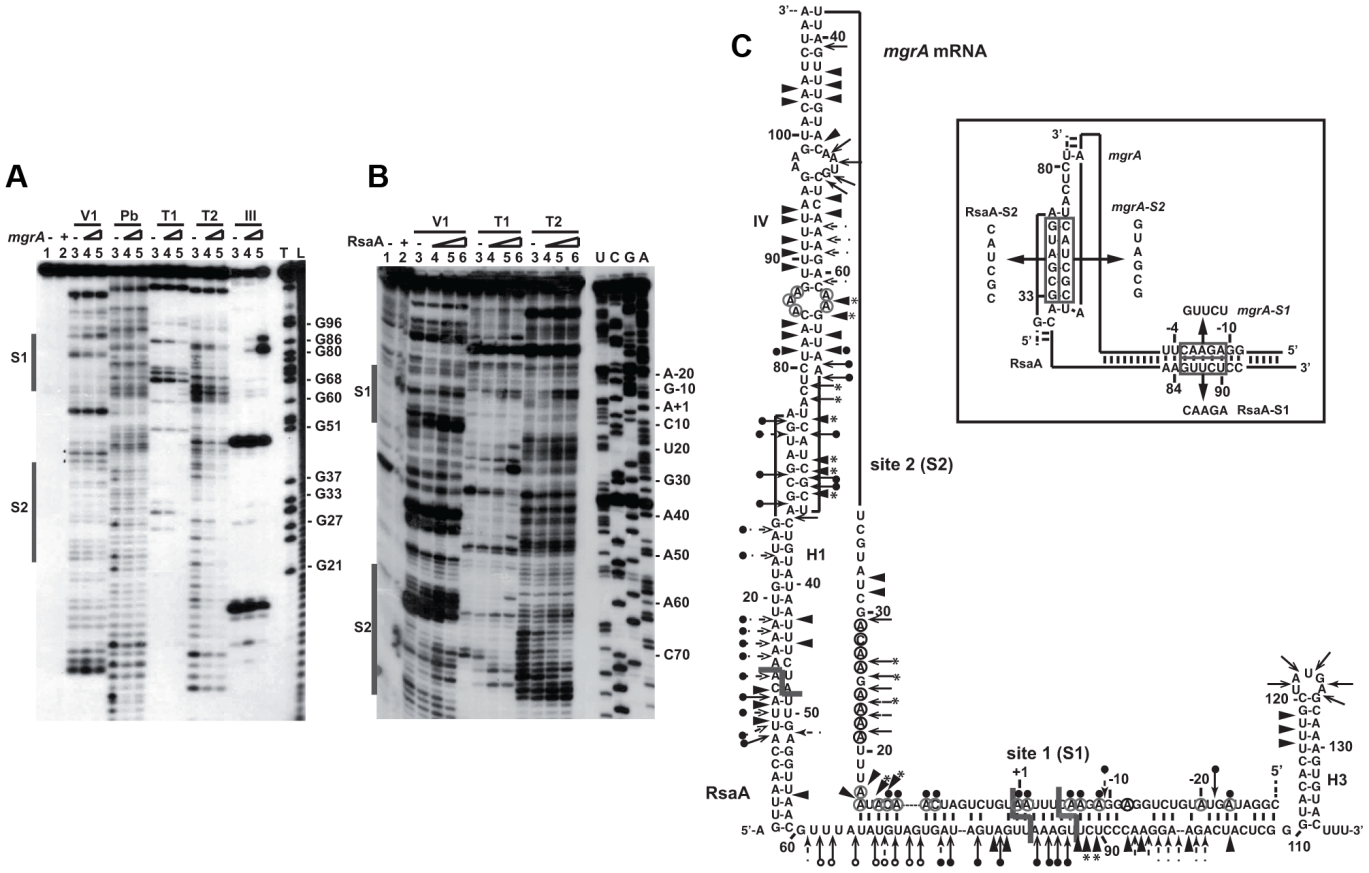
Probing the structure of the inhibitory RsaA-*mgr*A mRNA complex using enzymes and lead(II)-induced cleavages. (A) Reactions performed with 5′-end-labeled RsaA alone (lane 3) or in the presence of an excess of *mgr*A mRNA (lane 4, 25 nM; lane 5, 50 nM; lane 6, 250 nM). Lanes 1, 2: incubation controls on free RNA or bound to *mgr*A mRNA, respectively. The experiments were performed with RNase V1 (V1), lead(II) induced cleavages (Pb), Rnase T1 (T1), RNase T2 (T2) and the endoribonuclease III (RNase III). Lanes T, L: RNase T1 under denaturing conditions and alkaline ladders, respectively. The two regions protected by *mgr*A are shown by bars. (B) Enzymatic reactions performed on *mgr*A mRNA alone (lane 3) or bound to increasing concentrations of RsaA (lane 4, 25 nM; lane 5, 50 nM; lane 6, 250 nM). Same legend as in A. Lanes U, C, G, A: dideoxy-sequencing reactions. (C) Structure of the RsaA-*mgr*A mRNA complex. Summary of the enzymatic cleavages on RsaA, *mgr*A, and the complex. Enzymatic cleavages are given as follows: RNase T1 (black and plain arrow), RNase V1 (black and plain arrowhead), RNase T2 (arrow). Circled adenines and cytosines in *mgr*A mRNA are reactive towards dimethylsulfate modification. The RNase III cleavages led to RNA fragments, which terminated by a two-nucleotide 3′ overhang. Reactivity changes induced by the binding of RsaA are indicated as follows: black circles denote strong protection, enhancements and new RNase V1 cleavages are represented by asterisks. The mutations performed in RsaA and *mgr*A mRNA, are shown in the insert.

The predicted base pairing between RsaA (nts 65 to 108) and *mgr*A mRNA (nts −28 to +16) suggested that the RsaA-dependent repression of *mgr*A mRNA was governed by direct RsaA-mRNA pairing. To map more precisely the regions of interaction, we performed footprinting experiments using enzymatic and chemical probing. The regions of interaction between *mgr*A mRNA and RsaA were probed using RNase T1 (specific for unpaired guanines), RNase T2 (specific for unpaired nucleotides with a preference for adenines), RNase V1 and the endoribonuclease III (specific for double stranded regions), and lead(II)-induced cleavages (specific for unpaired nucleotides). We also used dimethylsulfate, which methylates adenines at the N1 position and cytosines at the N3 position to probe the structure of *mgr*A mRNA and its interactions with RsaA. The cleavage sites were analyzed using 5′ end labeled RsaA while for *mgr*A mRNA, the cleavages and chemical modifications were analyzed by primer extension using reverse transcriptase. Experiments are shown in Figures 2A–B.

The probing data obtained with RsaA alone correlated well with the existence of three hairpin loops (Figure S2A) as previously published [12]–[14], [19]–[23]. Indeed, single-strand specific RNase cleavages were mainly located in the apical loops and unpaired regions while RNase V1 cuts were predominantly found in the helices of RsaA. Furthermore, RNase III specifically cleaved helix H1, in agreement with the observation that RsaA decay is mediated by RNase III *in vivo* [23]. Enzymatic hydrolysis was then performed on 5′ end labeled RsaA incubated with increasing concentrations of *mgr*A mRNA. Binding of *mgr*A mRNA affected the cleavages at two distant regions of RsaA. Strong protections were observed close to the C-rich motif of RsaA, *i.e.* at A83–85 against RNase T2, at U82-A94 against lead(II)-induced cleavages, and at G86 against RNase T1 (Figure 2C). Concomitantly, several enhanced RNase V1 cuts were found at U88-C89, and two strong RNase III cleavages were observed at U82 and U87 of RsaA (Figure 2A). Unexpectedly, binding of *mgr*A mRNA also induced strong protections against single-strand specific probes in the hairpin loop H1 at residues A27 to G33 of RsaA revealing the existence of a second RNA binding site (Figure 2A, C).

The same experiments were performed on an *mgr*A fragment encompassing 124 nts upstream of the initiation codon and 258 nts of the coding sequence (Figure 2B). The enzymatic cleavages obtained on *mgr*A mRNA alone supported the existence of the long hairpin IV within the coding sequence as well as the stem-loop III structure, which partially sequestered the SD sequence (Figure S1B). The guanines of the Shine and Dalgarno (SD) sequence were indeed weakly accessible towards RNases T1 and T2. Binding of RsaA caused changes in the RNase cleavage patterns at two distant regions of the mRNA, namely around the RBS and in the hairpin loop IV located in the coding sequence (Figure 2C). In particular, strong RNase V1 cleavages occurred at positions C13 to A17, C70–C72 and C75, and two strong RNase III cleavages were observed at A+1 and C-5 of *mgr*A mRNA (Figure S2C). Concomitantly, RsaA-induced protections against RNase T2 were observed in the hairpin loop IV (at A67, A68, A74) and at nucleotide A-9 close to the SD sequence, and strong reduction of reactivities of adenines at N1 against dimethylsulfate were located at A+1, A-1, A-6, A-7 and A-9 in the RBS (Figures 2B, 2C). Interestingly, the hairpin loop IV carries the unpaired nucleotides UCGCUACU, which are fully complementary to the hairpin loop I of RsaA (AGUAGCGA) (Figure 2C).

All together, these data revealed that the *mgr*A mRNA-RsaA complex is composed of a bipartite site, which implies the formation of an imperfect duplex sequestering the RBS of *mgr*A and a loop-loop interaction. Furthermore, the long imperfect duplex creates a specific site for the RNase III.

### RsaA inhibits ribosome binding *in vitro* and regulates *mgr*A translation *in vivo*

**Figure 3:**
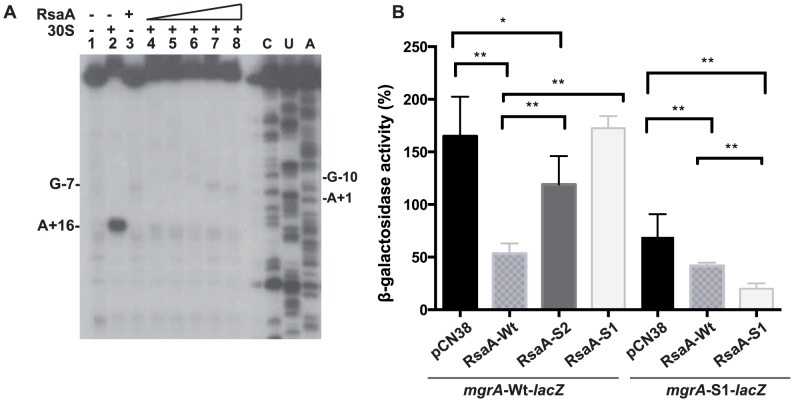
RsaA-dependent repression of *mgr*A mRNA translation. (A) Effect of RsaA on the formation of the initiation ribosomal complex. Lane 1: incubation control of *mgr*A mRNA; lane 2: formation of the initiation ribosomal complex containing *mgr*A mRNA, the initiator tRNA, and the 30S subunits; lane 3: incubation control of *mgr*A mRNA bound to RsaA; lanes 4 to 8: formation of the initiation ribosomal complex in the presence of increasing concentrations of RsaA: 20, 50, 150, 500 nM and 1 µM, respectively; lanes C, U, A: sequencing ladders. (B) ß-galactosidase activity detected from different gene fusions. ß-galactosidase activity was measured from P*rpo*B*-mgr*A (+1/+258)::*lac*Z fusion or from P*rpo*B*-mgr*A*-S1* (+1/+258)::*lac*Z fusion expressed in HG001-Δ*rsa*A strain. The experiments were carried out in Δ*rsa*A-HG001 mutant strain transformed with the plasmid pCN38 containing no insert (pCN38) or expressing the WT RsaA, or its two mutant derivatives RsaA-S1 (85GUUCU89 changed to CAAGA) and RsaA-S2 (28GUAGCG33 changed to CAUCGC). The ß-galactosidase activity was normalized for total cell density and is represented as a percentage of the uninhibited control. The results represented a mean of four independent experiments. Statistical differences * (p<0.05) and ** (p<0.005) between each construct were obtained by using Anova test.

Structure probing strongly suggested that RsaA would repress translation initiation of *mgr*A. Toeprinting assays were first used to monitor the effect of RsaA binding on the formation of the initiation ribosomal complex involving the 30S ribosomal subunits, the initiator tRNA^Met^, and *mgr*A mRNA. Formation of the ternary complex, which blocked the elongation of a cDNA primer by reverse transcriptase, produced a toeprint at A+16 (Figure 3A). Binding of RsaA completely abolished the toeprint at a ratio of 1∶1 (RsaA:*mgr*A mRNA). These data indicate that the RsaA-*mgr*A mRNA complex is rapidly formed and sufficiently stable to prevent the formation of the ribosomal initiation complex.

To further validate the *in vivo* relevance of RsaA-dependent repression of *mgr*A mRNA, we analyzed the expression of a reporter construct in *S. aureus* HG001-Δ*rsa*A strain expressing the wild-type (WT) RsaA. Mutations were introduced in both *mgr*A and RsaA at the two binding sites (S1 and S2), and were designed to disrupt or restore base pairings (Figure 2C). The entire leader regulatory region of the *mgr*A gene produced from P1 (124 nts upstream of the initiation codon) including 258 nts of the coding sequence was cloned in-frame with the *lac*Z gene into the pTCV-*lac* shuttle vector (Table S2). This construct is under the control of a constitutive promoter (P*rpo*B). ß-galactosidase activity assays were first performed in the *S. aureus* Δ*rsa*A mutant strain transformed with the pCN38 vector expressing WT RsaA from its own promoter. The ß-galactosidase activity was 4-fold lower in the Δ*rsa*A mutant strain expressing WT RsaA than in the same strain transformed with the control vector (Figure 3B). To probe the importance of the first binding site, we introduced mutations in the stem-loop structure H2 of RsaA where 86GUUCU90 was substituted by CAAGA (RsaA-S1) to weaken the interaction with the RBS of *mgr*A mRNA (Figure 2C). The ß-galactosidase activity was reproducibly enhanced 3-fold in the Δ*rsa*A mutant strain expressing RsaA-S1 as compared to the Δ*rsa*A strain expressing WT RsaA (Figure 3B). Thus, disruption of the predicted base pairings alleviated the repression of the reporter *mgr*A*-*WT*-lac*Z gene. We then introduced changes in the RBS of *mgr*A*-lac*Z reporter gene where -9AGAAC-5 was replaced by UCUUG to restore base pairing with RsaA-S1. This mutation weakened the strength of the SD-aSD helix since the first adenine of the SD sequence was substituted by a guanine. Indeed, the resulting mutated *mgr*A-S1*-lac*Z fusion produced less ß-galactosidase than the WT fusion (Figure 3B). The expression of WT RsaA reduced only slightly the ß-galactosidase synthesis while RsaA-S1 caused a significant reduction of the ß-galactosidase synthesis from the fusion *mgr*A-S1*-lac*Z by a reproducible 4-fold factor (Figure 3B). The contribution of the second binding site in regulation was also analyzed. Mutations were introduced in the hairpin loop H1 of RsaA (RsaA-S2, 28GUAGCG33 changed to CAUCGC) to destabilize the loop-loop interaction. The data showed that the ß-galactosidase synthesis obtained from *mgr*A-WT-*lac*Z was strongly enhanced in the Δ*rsa*A strain expressing RsaA-S2 as compared to the same strain expressing the WT RsaA. Hence, disruption of the loop-loop interaction significantly alleviated repression of *mgr*A (Figure 3B).

Taken together, these data showed that RsaA represses the synthesis of MgrA at the post-transcriptional level through the formation of an RsaA-mRNA duplex, which sequesters the RBS of *mgr*A mRNA. Furthermore, the two distant regions of interaction contribute to the repression of MgrA synthesis.

### The two binding sites are essential for RsaA-dependent translation inhibition of *mgr*A

**Figure 4:**
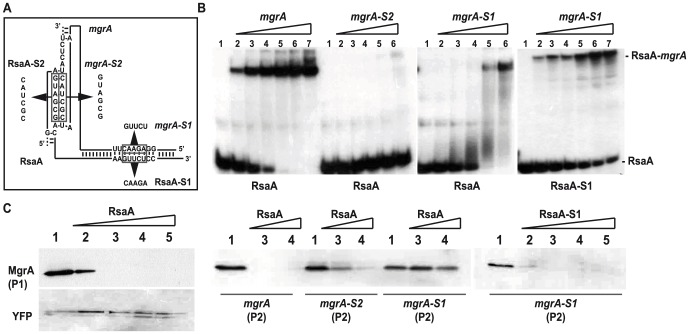
Contribution of the two binding sites of RsaA-*mgr*A duplex on translation repression. (A) Schematic drawings showing the two sites of interaction in the RsaA-*mgr*A complex. The various mutations are indicated. (B) Gel retardation assays to follow the formation of RsaA-*mgr*A complex. The 5′ end-labeled wild-type (WT) RsaA or the mutant RsaA-S2 were incubated with increasing concentrations of either the WT *mgr*A mRNA or its two mutant derivatives (*mgr*A-S1, *mgr*A-S2): lane 2, 5 nM; lane 3, 10 nM; lane 4, 20 nM; lane 5, 50 nM; lane 6, 100 nM; lane 7, 500 nM. Lane 1: free 5′ end-labeled RsaA. The bands corresponding to the free RsaA and to the complexes are specified. (C) *In vitro* translation assays using the PURESYSTEM. The reactions were performed with 10 pmol of WT *mgr*A or its two mutant derivatives (*mgr*A-S1, *mgr*A-S2) alone (lane 1) or in the presence of increasing quantities of WT RsaA or RsaA-S1 (lane 2: 5, lane 3: 10, lane 4: 30 and lane 5: 50 pmoles). The two mRNAs produced from the P1 or the P2 promoter were also analyzed. The same experiment was performed with YFP protein as the negative control. The proteins were separated on a SDS-PAGE 10% and were revealed using FLAG-specific antibodies.

The effects of mutations that disrupt or restore base pairings were further analyzed for their ability to form RNA-RNA interactions and their ability to inhibit *mgr*A translation. The formation of the RsaA-mRNA complex was followed using gel retardation assays (Figure 4B). *In vitro* 5′ end-labeled RsaA was incubated with increasing concentrations of *mgr*A mRNA. This experiment showed that *mgr*A mRNA binds to RsaA with a K_d_ value of around 20 nM. The initial rate of RsaA binding was estimated from a time-course analysis and resulted in an association rate constant of 5×10^5^ M^−1^s^−1^ indicating that the complex is rapidly formed. Mutations of the RBS of *mgr*A (*mgr*A-S1) had a strong effect on binding to WT RsaA while compensatory mutations performed in RsaA-S1 restored the binding albeit with less efficiency (K_d_ value around 50 nM). Disruption of the loop-loop interaction caused a strong effect on the binding of *mgr*A-S2 to WT RsaA (Figure 4B). Therefore, both regions of interaction contribute to the stability of the RsaA-mRNA complex indicating that they act in a cooperative manner.

We then performed *in vitro* translation assays (PUREsystem) using the whole *mgr*A mRNA (expressed either from the P1 or the P2 promoter) carrying the sequence encoding a FLAG tag to visualize the protein by Western blot using FLAG-specific monoclonal antibodies. Addition of WT RsaA specifically repressed the translation of both *mgr*A mRNAs (Figure 4C). These data were well correlated with the *in vivo* reporter assays (Figure 3B). The same experiment was performed with mutated versions of *mgr*A mRNA (*mgr*A-S1, *mgr*A-S2) expressed from the P1 promoter. Consistent with the binding experiments, the addition of WT RsaA had no effect on the translation of *mgr*A-S1 while RsaA-S1 efficiently inhibited the translation of *mgr*A-S1 (Figure 4C). Furthermore, destabilization of the loop-loop interaction had a significant effect on translation repression because higher concentrations of WT RsaA were required to achieve inhibition with *mgr*A-S2 compared to WT *mgr*A (Figure 4C).

All in all, these data show that RsaA inhibits initiation of translation of *mgr*A and that the two regions of interaction contribute to binding and regulation.

### Deletion of RsaA reduces biofilm formation

We then analyzed whether RsaA is able to counteract the action of the master regulator of transcription MgrA. The effect of RsaA was monitored on biofilm and capsule formation, which are two processes known to be regulated by MgrA [24], [26], [32], [33].

**Figure 5:**
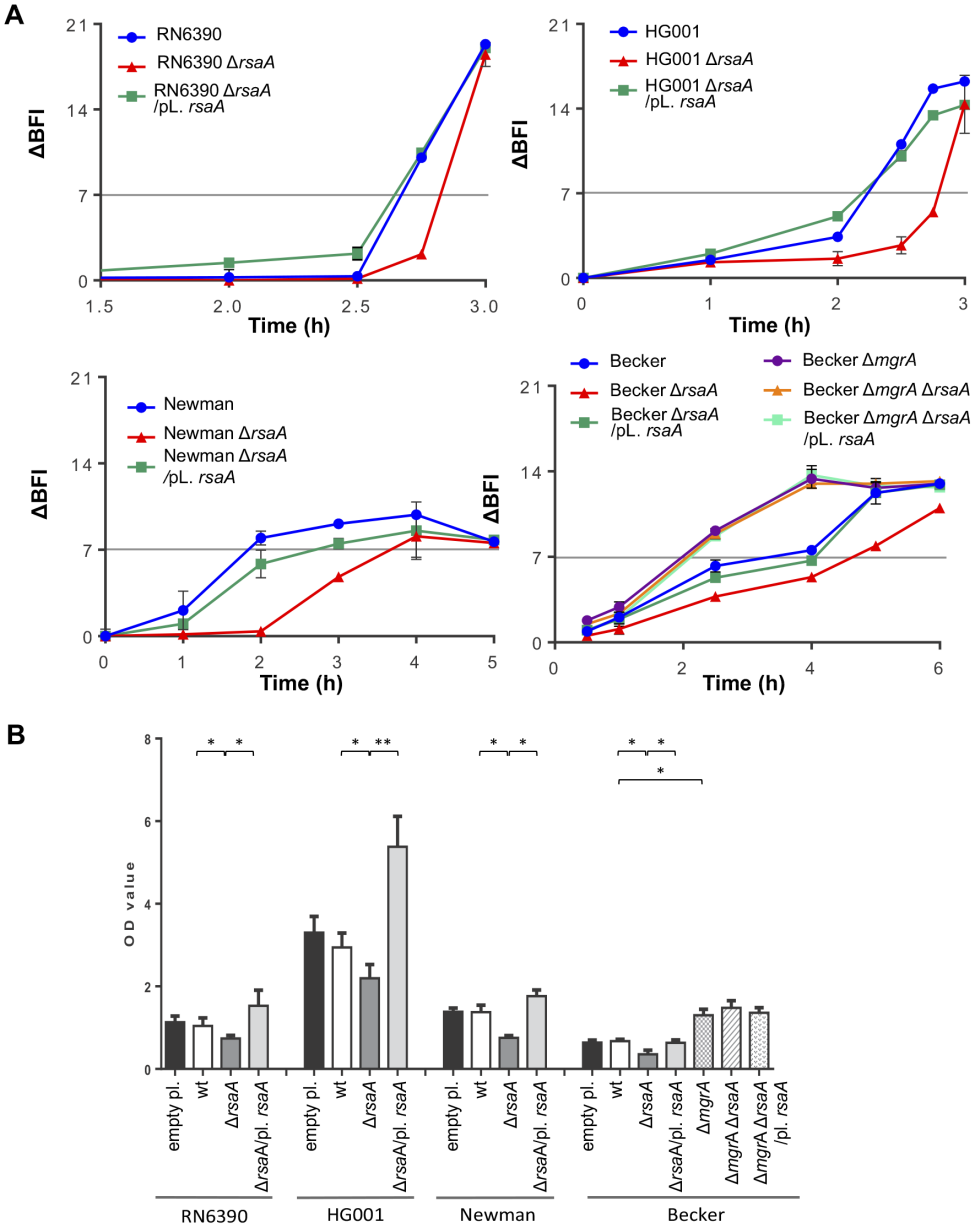
Effect of RsaA on biofilm formation. (A) Kinetics of the early phase of biofilm formation in different *S. aureus* backgrounds with the Biofilm Ring Test at different incubation times. Standardized bacterial cultures were incubated in 96 well micro-titer plate in the presence of magnetic beads at 37°C. A plate reader measures the number of beads in each well before and after magnetization. The beads are gathered in the center of the well if no biofilm is formed whereas the beads are dispersed if they are immobilized into the biofilm. The Biofilm Forming Index (BFI) corresponds to the mobility of beads under magnet action. Results are expressed as the ΔBFI corresponding to the difference between a control well (without bacteria) and the studied sample. A value of ΔBFI >7 means that the biofilm is formed. Data obtained for each strain were shown with a color code as given in the figure: blue for the parental wild-type (RN6390, Newman, Becker) strains, red for the Δ*rsa*A mutant strain, and green for the complemented strain expressing RsaA from a plasmid. (B) Analysis of the mature biofilm formation with crystal violet staining. Overnight bacterial cultures were standardized and incubated 24 h at 37°C in rich medium supplemented with glucose 0.25%. The mature biofilm was stained by crystal violet 0.1%, released with acetic acid 33%, and quantified by OD_570 nm_. The mean and the standard deviations are presented on the histogram. Statistical differences * (p<0.05) and ** (p<0.005) between each strains were obtained by using Anova test.

The biofilm Ring Test was used to follow the kinetics of early biofilm formation. This approach focuses on adhesion and aggregation of bacteria to a substrate (Figure 5A). We analyzed various strain backgrounds, namely RN6390 (σ^B−^), HG001 (σ^B+^), Newman and Becker (clinical isolates, σ^B+^) as well as their *rsa*A mutant and complemented counterparts. For all genetic backgrounds, the *rsa*A inactivation induced a delay in the kinetic of biofilm formation but this delay was variable according to the genetic background (Figure 5A). The delay in the kinetics of early biofilm formation of mutant vs WT strains was equal to 10 min for RN6390, 30 min for HG001, 1 h 30 for Becker and 2 h 10 for Newman. The *rsa*A complementation restored fully (RN6390 and HG001) or partially (Becker and Newman) the parental phenotype. The Becker Δ*mgr*A strain, generously provided by C. Lee, was used as a control. As expected [32], the inactivation of *mgr*A reduced the delay of early biofilm formation by about 1 h 30 in comparison to the WT strain. The double mutant Δ*mgr*A-Δ*rsa*A Becker strain did not further reduce the delay of biofilm formation, supporting that RsaA regulated the biofilm formation *via mgr*A inhibition. Of note, RsaA plasmid complementation of the Δ*mgr*A-Δ*rsa*A Becker strain did not change the biofilm phenotype obtained with the double mutant.

Quantification of mature biofilm after 24 h was also performed for all strains (Figure 5B). The level of mature biofilm was extremely abundant for HG001, intermediate for Becker and RN6390, and moderate for Newman. The inactivation of *rsa*A was associated with a significant decrease in biofilm thickness in the three strain backgrounds. Conversely, the plasmid complementation restored the parental WT phenotype and biofilm formation was even slightly higher due to the overexpression of RsaA. As a negative control, transformation with the empty plasmid showed no effect (Figure 5B). Furthermore, *mgr*A inactivation in the Becker strain induced a high increase in mature biofilm formation, as previously published [32]. As expected, the double mutant Δ*rsa*A-Δ*mgr*A Becker strain, and the same strain complemented with a plasmid expressing RsaA, produced a phenotype similar to that of the single Δ*mgr*A Becker strain.

The *ica*ADBC operon encoding the extracellular polysaccharide adhesin PIA/PNAG (polysaccharide intracellular adhesion/poly-N-acetylglucosamine) is responsible for some of the biofilm-positive phenotypes in *S. aureus* [34]. However, *S. aureus* can produce an alternative *ica*-independent biofilm *via* the expression of *mgr*A [32]. To rule out an involvement of *ica*ADBC in the RsaA-dependent biofilm formation, we quantified PIA/PNAG production using an anti-PIA/PNAG specific antibody in the three strain backgrounds. We saw that the inactivation of *rsa*A had no effect on PIA/PNAG production for the equivalent quantity of bacterial lysates (Figure S3).

In summary, these data suggested that RsaA is acting upstream of MgrA and activates biofilm formation through the inhibition of MgrA synthesis.

### RsaA inhibits capsule production and prevents opsonization of *S. aureus*

**Figure 6:**
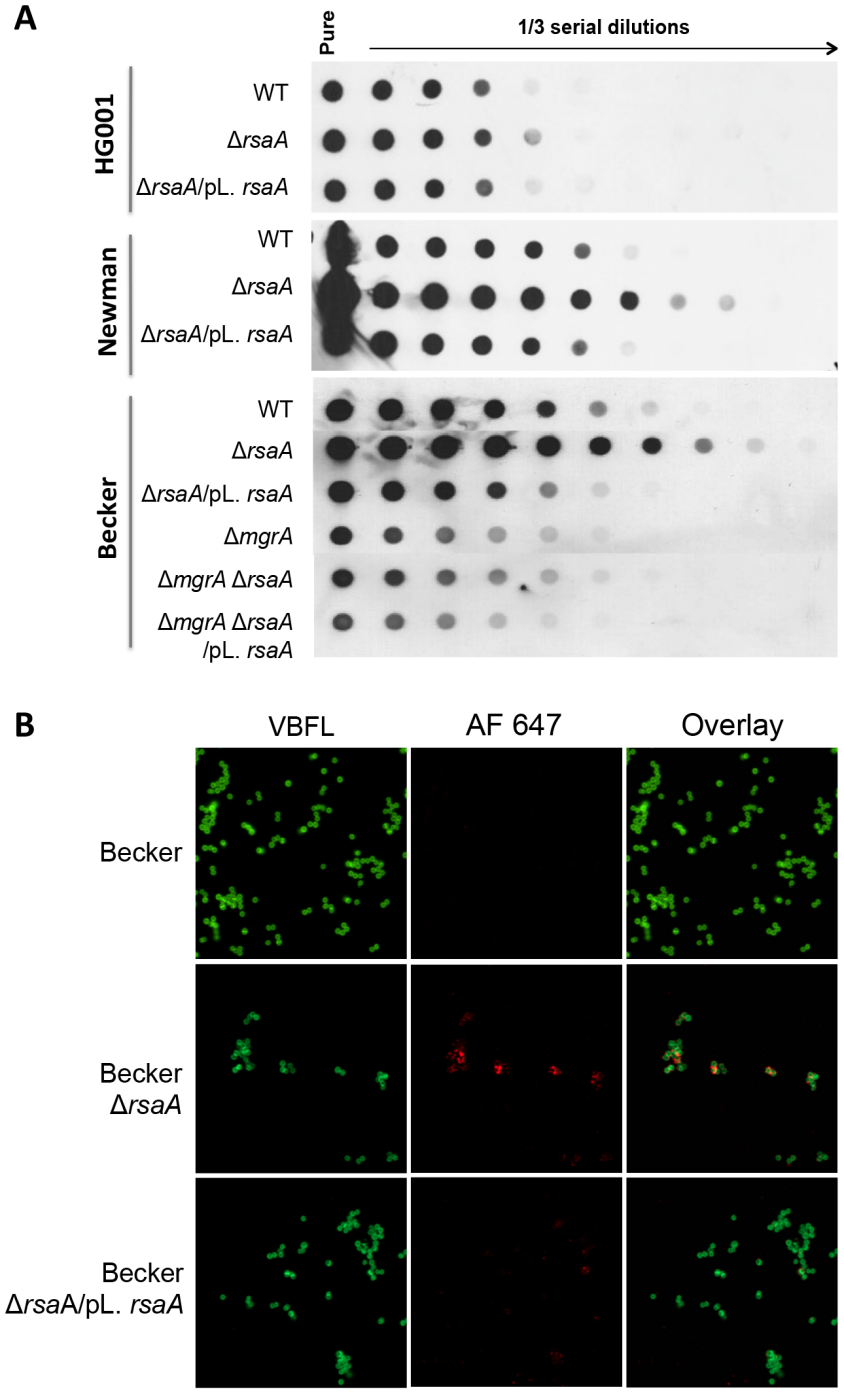
Effect of RsaA on capsule formation. (A) Dot-blot analysis of capsular polysaccharide accumulation in wild-type strains and its corresponding Δ*rsa*A mutant and complemented strains after growth in BHI for 16 h. Standardized bacterial suspensions were treated with lysostaphine, DNase I and proteinase K, and 1/3 serial dilutions were spotted onto nitrocellulose membranes. Capsular polysaccharides were detected with an anti-CP5 (HG001 and Newman) or CP8 (Becker) antisera. The Δ*rsa*A mutant produced a higher level of capsular polysaccharides and the Δ*mgr*A mutant produced a lower level of capsular polysaccharides. The inactivation of both *rsa*A and *mgr*A revealed that the repression of capsule production by *rsa*A achieved via the repression of *mgr*A. (B) Capsule production in Becker strain and its derivatives grown for 7 h in BHI at 37°C. CP8 expression was determined by indirect immunofluorescence of WT strain, the Δ*rsa*A mutant strain (LUG2010) and the mutant strain complemented with a plasmid expressing RsaA (LUG2032). The bacteria were labeled with Bodipy-vancomycine and capsule was labeled with an anti-CP8 antibody and a rabbit anti-IgG coupled with a fluorochrome AlexaFluor 647.

To study whether RsaA was involved in capsule regulation, we quantified capsule production after an overnight culture using anti-CP5 or anti-CP8 specific antibodies. Using equivalent amounts of bacterial lysates, we found that the Becker strain (capsular polysaccharide type 8, CP8) produced more capsular polysaccharides than Newman and HG001 (capsular polysaccharide type 5, CP5). The inactivation of *rsa*A induced a significant increase in CP8 biosynthesis in the Becker strain and in CP5 in the Newman strain while only a slight increase in CP5 was detectable for HG001 (Figure 6A). The expression of RsaA from a plasmid restored the parental WT phenotype for all strains. The same results were obtained after 6, 9 and 12 h of culture (data not shown). To verify that the regulation of capsule production by RsaA resulted from *mgr*A repression, we again analyzed the phenotype of the double mutant Δ*rsa*A-Δ*mgr*A in the Becker strain background. Compared to the WT, the level of CP8 production was considerably reduced in the single Δ*mgr*A mutant strain as well as in the double mutant Δ*rsa*A-Δ*mgr*A strain. Noticeably, we reproducibly found that the complementation of the double mutant with a plasmid expressing RsaA further reduced the level of CP8 production, suggesting the existence of an *mgr*A-independent repression effect of capsule production. This additional effect might be attributed to the RsaA-dependent inhibition of SpoVG (Figures 1, S1), a protein involved in the capsule formation [29].

To illustrate the accumulation of capsular polysaccharides in Δ*rsa*A mutant strains, indirect fluorescence was performed to investigate the capsule formation of the Becker strain and its derivatives after 7 h of growth in BHI. Strikingly, the capsule was highly labeled only in the Δ*rsa*A mutant Becker strain contrasting with the WT parental and complemented strains (Figure 6B). To verify the specificity of the secondary antibody, the same experiment was performed without the primary CP8 antibody, and as expected, all strains were only labeled in green (data not shown).

Because capsular polysaccharides protect *S. aureus* against opsonophagocytic killing by polymorphonuclear leukocytes (PMNs) [35], [36], we measured the percentage of bacteria killed after contact with serum and PMNs in strains expressing or deleted for RsaA. The data were not statistically significant because a large variability of bacterial survival was observed due to inter-PMNs donor differences. However, in the mutant strains, fewer bacteria were killed than in the parental strains expressing RsaA (particularly in the Becker strain background) in each replicate (Figure S4).

These data showed that RsaA significantly represses capsule synthesis in all strain backgrounds and renders *S. aureus* more sensitive to opsonophagocytic killing by PMNs.

### RsaA attenuates virulence in mice infectious models

Because RsaA impacts both biofilm and capsular formation, we selected two animal models in mice that were considered relevant with respect to these processes, namely, the catheter infection model (for biofilm) and the intraperitoneal infection model (for the capsule).

**Figure 7:**
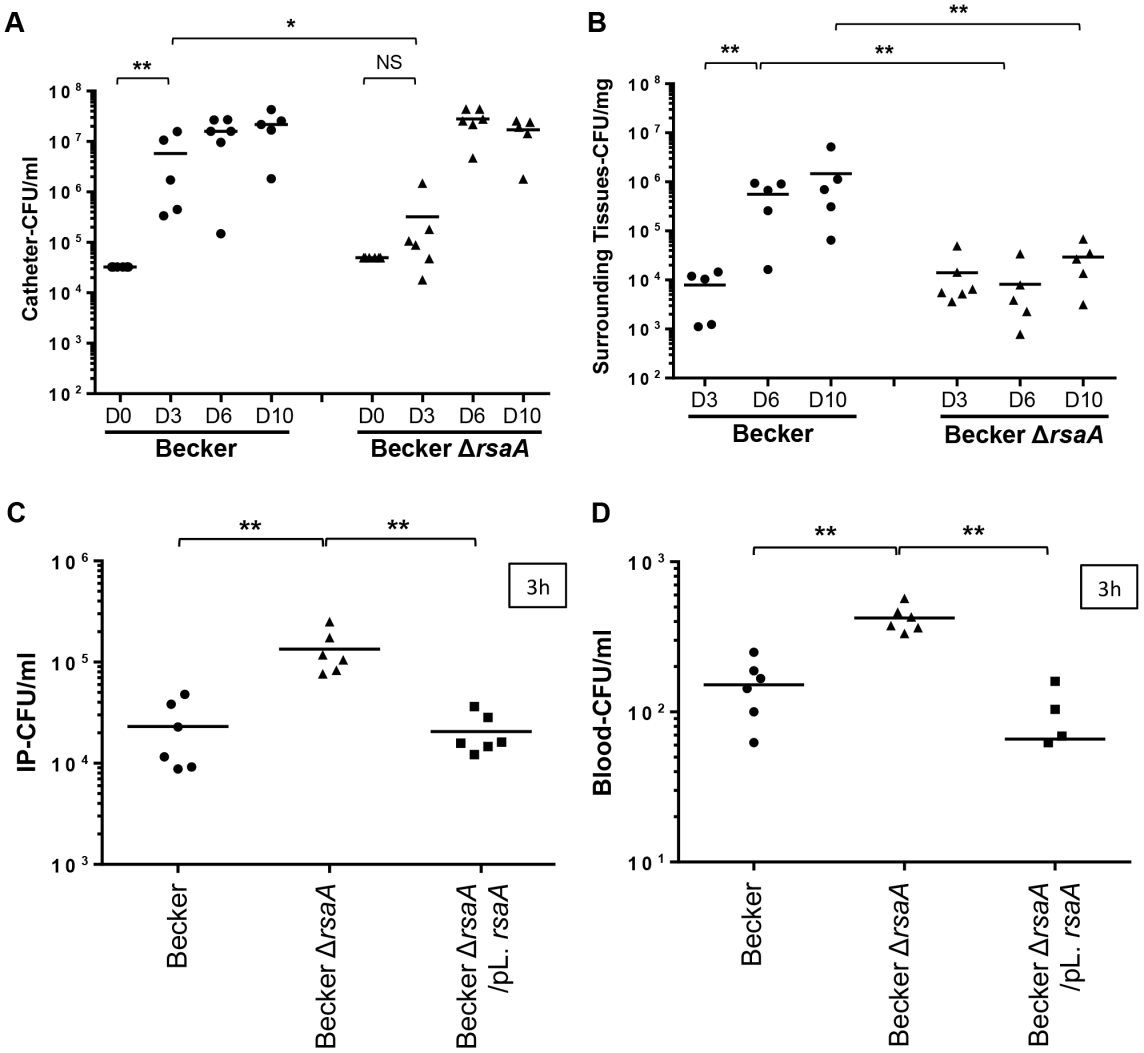
The impact of RsaA on virulence in *in vivo* animal models. Two *in vivo* infections models were developed: a catheter infection model (to mimic a chronic infection) and a bacteremia model (to mimic an acute infection). Quantification of bacteria into catheter (A) and in the surrounding tissues (B) after 3, 6 and 10 days post-infection. After euthanasia of mice, the catheters and surrounding tissues were removed and processed as described in Materials and Methods. Bacteria were enumerated by serial dilution and plate counting on agar plates. The results were expressed in number of CFU/ml. Each point designates the number of bacteria for each mouse and the black line represents the mean of each tested condition. The second model was performed by intraoperitonnal (IP) injection of 1×10^6^ bacteria. At 3 h post-infection, viable bacteria present into IP (C) and in blood (D) were enumerated by serial dilution and plate counting on agar plates. The results were expressed in number of CFU/ml. Statistical differences * (p<0.05) and ** (p<0.005) between each groups were obtained by using Mann Whitney test.

For the *in vivo* catheter infection model, sections of polyurethane catheter (1 cm) were implanted sub-cutaneously into C57BL/6 mice and filled with about 4×10^4^ bacteria/25 µl (Becker WT and Δ*rsa*A mutant strains) in PBS (6 mice/group). The catheters were implanted for 3, 6 or 10 days. No difference in weight was observed between the mice infected by the WT and mutant strains (data not shown). Both strains led to a local infection with swelling but the size of the swelling was not significantly different between the studied strains (data not shown). At day 3, the concentration of colony-forming units observed on the catheters was significantly higher in mice infected with the WT strain than those infected by the Δ*rsa*A mutant strain (p = 0.0173) (Figure 7A). Between days 6 and 10, this difference was no longer observed in mice infected with WT and mutant strains, likely due to the saturation of the growth within the catheters (Figure 7A). However, between days 3, 6 and 10, more colony-forming units were observed in the surrounding tissues of the mice infected with the WT strain while the number of colony-forming units of the mutant Δ*rsa*A strain remained unchanged (Figure 7B).

For the bacteremia model, we included the two major capsular types of *S. aureus* (type 5 and 8), namely Newman (type 5) and Becker (type 8), their Δ*rsa*A mutant derivatives, as well as their complemented counterparts (studied at the early time point only). Mice were challenged with sub-lethal doses of each strain by intra-peritoneal injection (1×10^6^ bacteria/500 µl). Animals challenged with the strongly encapsulated strains (Becker-Δ*rsa*A) showed a significantly (p<0.005) higher bacteremia level at 1 h and 3 h after inoculation compared with animals infected with the parental and complemented strains (both less encapsulated) (Figures 7C and S5A). Furthermore, after 1 h and 3 h, the blood concentrations of Δ*rsa*A bacteria were significantly higher (p<0.05) than those of parental and complemented strains (Figures 7D and S5B). The same results were obtained with Newman background (Figures S5C–F). Evolution of the infection was followed by collecting the mice spleen for bacterial numeration. After 1 and 2 days, the bacterial counts in the spleen were generally higher in the Δ*rsa*A mutant strain- vs WT strain-infected animals for both Becker (Figure S5H) and Newman (Figure S5G) backgrounds. However, this difference only reached significance in the spleen after 1 day with Newman strain and after 2 days with Becker strain.

Our data obtained with both animal models suggested that RsaA diminishes invasiveness but favors chronic infections and local colonization.

### RsaA is expressed by clinical isolates of *S. aureus*

The observation that RsaA impacted oppositely biofilm formation and invasiveness raised the question as to whether clinical isolates would express differential levels of RsaA depending on the clinical situation in which they had been sampled. To this end, a series of 18 isolates reflecting the commensal status of *S. aureus* (nasal colonization) as well as *S. aureus* diseases in various forms (chronic lung infection in cystic fibrosis patients, non-invasive acute skin and soft tissue infections, severe invasive disease illustrated by community-acquired pneumonia and infective endocarditis), were tested for RsaA expression at mid and late-exponential phases by RT-qPCR. The results revealed no specific correlation between RsaA expression levels and invasiveness, both at the mid and late-exponential state of growth (Figure S6A). Of note, all reference strains tested (HG001, Becker, Newman, USA300 LAC and SF8300, and the European CA-MRSA ST80) showed equivalent levels of RsaA production (Figure S6B).

## Discussion

### RsaA regulates the synthesis of the master global regulator MgrA

The virulence of *S. aureus* is in part conferred by numerous effector proteins that interact with the host. The levels of these virulence factors are controlled by a set of more than 20 transcriptional regulatory proteins [2]. However, *S. aureus* strains are genetically highly diverse and it is well recognized that their regulatory networks are not uniform. In order to determine the virulence traits of a defined strain, we must understand how the regulators interact with each other and how novel regulators such as non-coding RNAs influence these networks.

**Figure 8:**
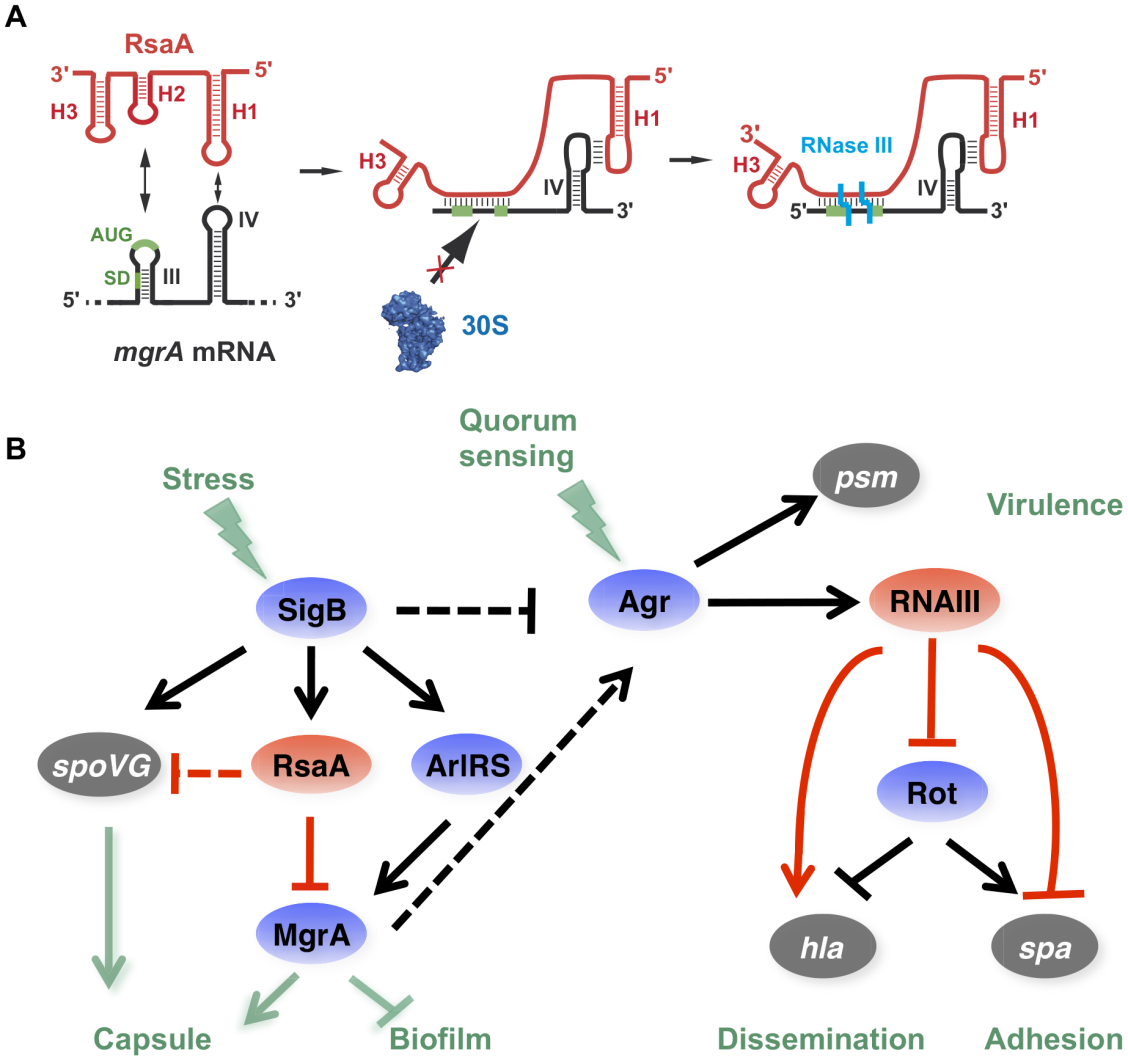
RsaA and its regulatory circuits. (A) Schematic drawing summarizing the regulatory mechanism. RsaA binds to *mgr*A mRNA and inhibits translation by preventing the 30S subunit binding, and recruits RNase III to induce simultaneous degradation of both RNAs. (B) RsaA is activated by σ^B^ and in turn represses *mgr*A translation. RsaA is thus indirectly linked to RNAIII regulatory networks because MgrA activates *agr*ACDB expression while σ^B^ represses it [32], [41]. Arrows are for activation, bars for repression. In blue are the transcriptional protein regulators, in red the regulatory RNAs and in grey the virulence factors. Red lines corresponded to post-transcriptional regulation and black lines to transcriptional regulation. The regulatory events for which direct regulation is not yet demonstrated are shown by dotted lines.

In this work, we show that the small non-coding RNA RsaA functionally links two global regulators, the σ^B^ and MgrA proteins. The transcription of RsaA was shown to be under the control of σ^B^ protein [20], while its fate was determined by two endoribonucleases, the double-strand specific RNase III [23] and RNase Y [37]. Our data indicate that the *mgr*A mRNA is repressed at the stationary phase of growth in an RsaA-dependent and RsaA-independent manner. The *mgr*A mRNA is transcribed from two promoters, and the mRNA with a longer 5′UTR was no longer produced at the late-exponential phase when RsaA was produced (Figure 1F). Whether the regulation is mediated by a protein- or another sRNA remains to be studied. Using a combination of approaches *in vivo* and *in vitro*, we showed that the primary effect of RsaA is to repress the translation of *mgr*A mRNA by preventing the formation of the ribosomal initiation complex. The steady state level of the mRNA was also substantially enhanced in the Δ*rsa*A strain suggesting subsequent degradation of the repressed mRNA. A reduction of *mgr*A mRNA from the P1 promoter was also observed at the post-exponential phase of growth in various σ^B+^ strains, and the levels of *mgr*A mRNA were found to be higher in RN6390 (σ^B−^) than in SH1000 (σ^B+^) [38]. This was correlated *in vivo* with the level of MgrA protein, which was found to be higher in the Δ*rsa*A mutant strain than in the isogenic WT strain (Figures 1C, D). *In vitro* binding assays showed that RsaA binds to *mgr*A mRNA with a fast association rate constant, which is essential for efficient repression because the sRNA has to bind to its target mRNA within a short time frame before the stable ribosomal initiation complex is formed [39]. Two stretches of highly conserved and unpaired nucleotides of RsaA bind to two distant regions of *mgr*A, a C-rich motif of RsaA masks the SD sequence of *mgr*A while a loop-loop interaction occurs between two hairpin loop structures found in the 5′ end of RsaA and in the coding sequence of *mgr*A (Figure 8A). Mutational analysis revealed that the two RsaA binding sites are required for optimal binding and *mgr*A translation repression *in vivo* (Figures 3 and 4).

It is worth noting that RsaA and RNAIII share very similar regulatory mechanisms [11], [40] suggesting that the importance of bi-partite RNA-RNA binding sites in translation repression appears to be more general than previously expected in *S. aureus* (Figure 8A). In both cases, their mRNA targets are characterized by two regulatory modules: the SD sequence interacts with a C-rich motif within the regulatory RNAs to prevent ribosome binding and to create a specific RNase III binding site, while a hairpin motif constitutes either a second RNA binding site to stabilize the RNA-RNA interaction or to promote a specific binding site for RNase III [5], [10]. For the RsaA-*mgr*A complex, the second interaction site involves a loop-loop interaction, which greatly enhances the stability of the complex and the efficiency of translation repression (Figure 4). RNase III-dependent cleavages were observed at the site of interaction formed between the RBS of *mgr*A and the C-rich motif of RsaA. Note that *mgr*A mRNA and RsaA were co-immunoprecipitated with RNase III and the half-life of RsaA was significantly enhanced in the Δ*rnc* mutant strain [23]. As it was shown for RNAIII, it is tempting to propose that RNase III also contributes to the degradation of the translationally repressed *mgr*A mRNA. In addition, since MgrA positively autoregulates its own transcription [27], RsaA-dependent repression of *mgr*A translation might indirectly inhibit *mgr*A transcription.

### RsaA-dependent regulatory circuit might be linked to the quorum sensing system

By regulating the translation of MgrA, RsaA participates in a complex regulatory network involving two global regulators, which modulate the levels of RNAIII, thus affecting biofilm formation and capsule synthesis (Figure 8). RsaA expression is positively regulated by the alternative σ^B^ factor [20], an observation that is well correlated with our data. Indeed, *rsa*A inactivation affected less strongly the kinetics of biofilm development in RN6390 (σ^B−^, 10 min) than in HG001 (σ^B+^, 30 min) due to the fact that the sRNA was not strongly expressed in RN6390 [20]. RsaA synthesis follows the same pattern as σ^B^, which is activated at a basal level at the exponential phase and strongly enhanced at the stationary phase [41]. Furthermore, σ^B^ affected the transcription of the virulence genes in an opposite manner to RNAIII, and this effect would be in part the consequence of lower RNAIII levels in strains harbouring a fully active σ^B^ [41]. σ^B^ was also shown to enhance resistance to oxidative and UV stresses, biofilm, and capsule formation. The σ^B^-dependent activation of biofilm formation occurs *via* the *ica*ADBC operon [42], [43]. We show that RsaA does not perturb the production of the PIA/PNAG polysaccharides produced by the *ica*ADBC operon (Figure S2), but affects pathways that are predominantly dependent on *mgr*A. σ^B^ is also known to activate the capsule synthesis *via* the two-component system ArlRS and *yab*J-*spo*VG [33]. It is worth noting that *yab*J-*spo*VG is a potential mRNA target of RsaA whose expression was altered in the proteome analysis, and for which base pairings with RsaA have been predicted (Figure 1). The main target of RsaA, which is the transcription factor MgrA, regulates more than 350 genes [26]. MgrA is activated by the *arl*RS operon, whose expression is controlled by σ^B^ [26]. Taken together, the expression of MgrA is under the control of an incoherent feed-forward loop, involving a positive loop mediated through ArlRS and a negative loop mediated through RsaA (Figure 8). In addition, a recent study revealed that the DNA binding activity of MgrA is modulated through specific phosphorylation by the eukaryotic like kinase phosphatase pair Stk1-Stp1 [44]. Hence, the expression of this master regulator is controlled through multiple pathways. MgrA is central to the regulation of biofilm and represses the production of two out of the three components of the biofilm, i.e. surface proteins and exogenous DNA release [32]. Indeed, MgrA negatively regulates the DNA release by suppressing *cid*A expression and activating *lrg*AB encoding a peptidoglycan hydrolase and an inhibitor of murein hydrolase activity, respectively [32]. Through its synergistic action on these two loci, MgrA inhibits cell autolysis and the release of DNA within the biofilm [32]. The second control exerted by MgrA is to activate the *agr* system, in a quorum sensing-independent pathway, to inhibit the synthesis of surface proteins essential for adhesion [25], [26]. Although *mgr*A mutations significantly reduced RNAIII levels [25], [27], the protein also bypasses the quorum sensing system by directly activating the *hla* gene and repressing *spa* gene expression *via sar*S. As with the *agr* system, MgrA also has a positive effect on capsule synthesis [26], and in a rat infective endocarditis model, MgrA was shown to be the major regulator of capsule formation *in vivo* [28]. In correlation with these studies, we showed that inactivation of *rsa*A alleviates the repression of *mgr*A to increase capsule synthesis (Figure 6). Therefore, we propose that RsaA functionally links the global regulators σ^B^ and *mgr*A, with potential consequences on the temporal expression of virulence determinants ([25], [26]; Figure 8).

We demonstrate that RsaA activates biofilm formation (Figure 5) and represses capsule synthesis (Figure 6) most probably *via* the repression of *mgr*A translation. The regulation of these two phenotypes has been extensively studied in pathogenic bacteria demonstrating a complex interdependency. Studies in *Staphylococcus haemolyticus* and *Streptococcus pneumoniae* highlighted that encapsulated strains produced little or no biofilm, whereas non-capsulated strains produced a strong biofilm [45]. Moreover, inactivation of the *cap* operon of *S. haemolyticus* JCS1435 (sharing 57% of sequence similarity with *S. aureus cap)* induced biofilm formation [46] suggesting that the capsule would partially mask or inhibit the surface proteins essential for biofilm formation. In our study, we demonstrated this imbalance in favour of biofilm production for the HG001 strain that is minimally encapsulated but produces lots of biofilm; or in favour of the capsule synthesis for Becker and Newman strains that produce small amounts of biofilm but abundant capsule (Figures 5 and 6). The mechanistic details of this imbalance are not totally understood and may certainly involve other factors besides MgrA and RsaA.

### RsaA favors chronic infection and attenuates invasiveness

The pathogenic bacterial chromosome is composed of the core genome plus accessory elements. The accessory genome contains genes that have been horizontally acquired through mobile genetic elements (plasmids, phages, pathogenicity islands). The acquisition of these elements, which often encode virulence determinants and antibiotic resistance, has been accompanied by subsequent adaptation of the bacteria to successfully incorporate these novel elements [47]. Some pathogenic bacteria harbour genes that can hinder the expression of acquired virulence factors, and certain genes must be inactivated for the expression of full pathogenicity [48]. Other bacterial genes lead to hypervirulence when they are inactivated [49]. This family of genes was identified in several pathogenic bacteria such as *Listeria monocytogenes* [50] or *Salmonella enterica* [51]. For instance, in *S. enterica* the leucin-responsive regulatory protein (Lrp) represses key virulence factors involved in the invasion of host cells [51]. These genes encode proteins, but recently an antisense RNA in *S. typhimurium* was shown to kinetically control the virulence determinant MgtC, which is required for defense against the host immune system [52]. Deletion of this antisense RNA causes an hypervirulent phenotype. In the present study, we demonstrate that RsaA, a non-essential ncRNA present in all *Staphylococcus* species [20] reduces the virulence of *S. aureus*. Indeed, the deletion of *rsa*A increases the invasiveness of the bacteria in the mice sepsis model and resistance to opsonophagocytic killing. Conversely, RsaA promotes biofilm formation and catheter colonization in mice, potentially promoting harmful colonization and chronic infections in human. Noticeably, quantification of RsaA expression from human clinical isolates revealed that Rsa is functional in all clinical isolates of *S. aureus* studied so far [53] but is not differentially expressed depending on the level of invasiveness ranging from commensalism to deep-seated infections (Figure S6). Of note, we also showed previously by direct quantification from clinical samples that RsaA is expressed in all clinical samples including nasal secretion [53]. Since *Staphylococci* are mainly present as commensal bacteria of warm-blooded organisms (e.g. mammals and birds), the functionality of RsaA as a suppressor of virulence may have been positively selected through evolution because it is favorable for commensalism and saprophytic interactions with the host. We thus postulate that mutations leading to RsaA deficiency would be counter selective, perhaps because hypervirulence would be deleterious to the human host that is the natural reservoir of this bacterium.

## Materials and Methods

### Ethics statement

All mouse protocols were carried out in strict accordance with the Directive 2010/63/EU revising Directive 86/609/EEC on the protection of animals used for scientific purposes. This directive was translated in the French regulation as the Décrêt n°2013-118 of Feb 2013 under the jurisdiction of the Ministry of Education, Research and Technology. The protocols were approved by the CECCAPP (*Comité d'Evaluation Commun au Centre Léon Bérard, à l'Animalerie de transit de l'ENS, au PBES et au laboratoire P4)* under identification numbers ENS_2011_033 and ENS_2012_051 for biofilm and bacteremia models, respectively.

### Strains, plasmids and growth conditions

The *Staphylococcus aureus* strains, clinical isolates, and plasmids used in this study are listed in Table S2. *Escherichia coli* strain DH5α (Promega, Madison, USA) was used as a host strain for plasmid constructions. *S. aureus* strain RN4220 [54] was used as the recipient in electroporation of the constructed plasmids. *Staphylococci* were grown either on GP agar plates (1% peptone, 0,5% yeast extract, 0,5% NaCl, 0,1% glucose, 1,7% agar, pH 7,2) or in brain-heart infusion (BHI) supplemented with erythromycin (5 µg/ml) or chloramphenicol (10 µg/ml) when appropriate. *E. coli* strains were cultivated in Luria-Bertani medium (1% peptone, 0,5% yeast extract, 1% NaCl). Total DNA and plasmid DNA were prepared using DNAeasy tissue Kit Qiagen and Qiaprep Miniprep respectively (Qiagen, Valencia, USA). Transformation of *E. coli* DH5α was performed by thermic shock and *S. aureus* strains were transformed by electroporation (Bio-Rad gene Pulser).

The deletions/replacements Δ*rsa*A/*aph*A-3 mutants of *S. aureus* RN6390 (LUG1450), HG001 (LUG1630), Newman (LUG1680), Becker (LUG2010) and CYL1040 (LUG2009) strains were obtained using pMAD [55]. The kanamycin resistance gene *aph*A-3 was cloned in pMAD between two DNA fragments corresponding to the chromosomal regions upstream and downstream of the *rsa*A coding sequence using primers *rsa*A-76/*rsa*A-1120 and *rsa*A-1246/*rsa*A-2194, respectively (Table S3). The resulting plasmid (pLUG754) was electroporated into RN4220 recipient strain and then transferred to RN6390, HG001, Newman, Becker and CYL1040. Growth at non-permissive temperature (44°C) was followed by several subcultures at 30°C and 37°C to favor double crossing over as previously described [10]. To complement these mutant strains, the *rsa*A gene was amplified using *rsa*A-76/*rsa*A-2194 oligonucleotides and inserted into pLUG274 forming pLUG959. Complementation of mutant strains was performed using pE194, pCN51 or pCN38 carrying *rsaA* gene (Table S2). The CYL1040 was generously provided by CY Lee [24].

### Preparation of RNAs

PCR fragments containing RsaA and its derivatives (RsaA-S1, RsaA-S2) were cloned into pUC18 vector, and then were transcribed *in vitro* using T7 RNA polymerase after plasmid linearization. The WT *mgr*A mRNA (−124 to +258) and its derivative (*mgr*A-S1, *mgr*A-S2) fragments were transcribed from PCR fragments (Table S3). The transcribed RNAs were purified by 8% polyacrylamide-8 M urea gel electrophoresis. After elution in 0.5 M ammonium acetate/1 mM EDTA buffer, the RNAs were precipitated twice with ethanol. The 5′ end-labeling of dephosphorylated RNA or DNA oligonucleotides was performed with T4 polynucleotide kinase and [γ-^32^P]ATP. Before use, RNAs were renatured by incubation at 90°C for 2 min in the absence of magnesium and salt, 1 min on ice, followed by an incubation step at 37°C for 15 min in TMN buffer (20 mM Tris-acetate pH 7.5, 10 mM magnesium-acetate, 150 mM Na-acetate).

### Relative quantification of sRNA from *S. aureus* by RT-PCR

RNA extraction was performed from mid (OD_600_ = 0.5) or post-exponential phase (OD_600_ = 6) grown bacteria using RNeasy Plus mini kit (QIAGEN) as described [53]. One mg of total RNA was transcribed into cDNA using Reverse transcription system (Promega). Subsequently, the cDNA was used as a template for the real-time PCR amplification using a Realpex2 instrument (Eppendorf) and the specific primers shown in Table S1. The amplification products were detected using SYBR Green. The relative amounts of amplicons for each gene were determined using quantitative PCR relative to an internal standard (*gyr*B encoding Gyrase B subunit) as described [53]. The expression levels of the sRNA were expressed as n-fold differences relative to the calibrator.

### *In vivo* ß-galactosidase assays

Translation fusions were constructed with plasmid pLUG220, a derivative of pTCV-*lac*, a low-copy-number promoter-less *lac*Z vector (Table S2). The whole leader region of *mgr*A (nts −124 to +258) mRNA (from P1 promoter) including 258 nts of the coding sequence, was cloned downstream the *rpo*B promoter in frame with *lac*Z. ß-galactosidase activity was measured four times on duplicate cultures with the Enzyme Assay System (Promega).

### Northern blots

Electrophoresis of total RNA (10 µg) was done on a 1% agarose gel containing 25 mM guanidine thiocyanate and vacuum transfer to nylon membrane. Hybridizations with specific digoxigenin-labeled RNA probes complementary to *mgr*A mRNA or RsaA and luminescent detection were carried out as described previously [10]. For all experiments, we verified the quantity of 5S rRNA using a digoxigenin-labeled RNA probe.

### Gel retardation assays

Binding rate constant of RNAIII-*mgr*A mRNA complex was measured as described previously [10]. Binding of 5′ end-labeled *mgr*A mRNA to a ten-fold excess of unlabeled RsaA or its derivatives was performed at 37°C in TMN buffer. Samples were withdrawn at various time points (0–10 min), added to gel application buffer and loaded onto a native 5% polyacrylamide gel under non denaturing conditions. The gel was run at 4°C and constant voltage (300 V) for 3 h and subsequently dried. Bands corresponding to the RsaA-*mgr*A mRNA complex and free RsaA, respectively, were quantified using the SAFA algorithm. For determination of the dissociation rate constant of RsaA-*mgr*A mRNA complex, end-labeled *mgr*A mRNA was incubated with an increased concentrations of WT RsaA or its variants for 15 min at 37°C in TMN buffer. All experiments were done four times.

### Proteomics

Growth conditions, preparation of protein extracts, and separation of proteins using two-dimensional (2-D) gel electrophoresis concerted with protein identification by mass spectrometry approach have been described in [20]. Experimental details are given in the supplementary materials (Supplementary Protocol S1).

Relative MgrA protein expression level was defined by a spectral counting strategy (see Supplementary Protocol S1). Triplicate protein extracts from HG001 (WT), Δ*rsa*A mutant strain (LUG1630), and the same strain complemented with a plasmid expressing RsaA, were analyzed in separate LC/MS experiments and the MS/MS spectra number were compared for each protein. In parallel to MgrA, we analyzed several ribosomal proteins (L21, L22, L24) as internal controls of constitutively expressed proteins.

### Western blot analysis

Cell extracts from the post-exponential (OD_600_ = 4) phase of growth were prepared from various staphylococcal strains as described in Supplementary Protocol S1. The concentration of total proteins from clear lysates was determined using the Bio-Rad protein estimation kit and BSA as the standard. Equal amounts of total cellular proteins were separated on 15% polyacrylamide-SDS gels and transferred onto PVDF membranes. Blots were incubated at 20°C with an appropriate dilution (1∶1000) of anti-MgrA-specific monoclonal for 1 h (generous gift from A. Cheung, USA), followed by another 1 h incubation with a 1∶2500 dilution of goat anti-mouse peroxidase (HRP) conjugate. Prestained protein standards were used for molecular mass estimations, and the gel was stained by Coomassie blue to verify that the quantity of proteins was homogenous in each sample. Each experiment was repeated at least three times with different samples.

### RNA structure probing using enzymes

RNAIII-*mgr*A mRNA formation was carried out at 37°C for 15 min in TMN buffer. Enzymatic hydrolysis was performed either on cold *mgr*A (1 pmole) or 5′ end-labeled RsaA (50000 cpm) in 10 µl of TMN, in the presence of 1 µg carrier tRNA at 37°C for 5 min: RNase T1 (0.0025 units), RNase V1 (0.5 units), RNase T2 (0.05 U), RNase III (500 nM). Lead(II) induced cleavages were performed on 5′ end-labeled RsaA at 20°C in 20 µl of TMN buffer containing 2 µg of carrier tRNA with 2.5 µl of different concentrations of lead(II)-acetate (12, 40, and 80 mM) for 5 min at 37°C. The reactions were stopped with 5 µl of EDTA 0.1 M for lead(II)-induced cleavages and all other reactions were stopped by phenol extraction followed by RNA precipitation. The end-labeled RsaA fragments were sized on 12% polyacrylamide/8 M urea slab gels. For *mgr*A mRNA, the enzymatic cleavages were detected by primer extension with reverse transcriptase [56].

### Toeprinting assays

The preparation of the *S. aureus* 30S subunits, the formation of a simplified translational initiation complex with mRNA, and the extension inhibition conditions were described according to [57]. Experimental details are given in the supplementary materials (Supplementary Protocol S1).

### *In vitro* transcription-translation assays

The *in vitro* translation assays were carried out using the full-length WT *mgr*A or *mgr*A-S1 or *mgr*A-S2 mRNAs and the PURESYSTEMII classic kit (Cosmo Bio Co, Japon). All constructs carry an additional sequence corresponding to the Flag peptide, which was inserted at the 5′ end of *mgrA.* The reaction was done at 37°C for 1 h in the presence of 25 µl of the commercial solution A and 10 µl of the commercial solution B in the presence of 10 pmoles of mRNA. Experiments were also carried out in the presence of increasing concentrations of WT or mutant RsaA (5 to 30 pmoles). The tubes were placed on ice to stop the reactions, and the proteins were detected by western blot using antibodies against the FLAG tag.

### Biofilm Crystal Violet Staining and Biofilm Ring Test

The biofilm formation was detected by tissue culture plate method that allowed semi-quantitative measurement of biofilm formation as described previously by Heilmann *et al.* [58]. All tests were carried out three times and average OD value was calculated for each strain and for negative controls. Experimental details are given in the supplementary materials (Supplementary Protocol S1).

The kinetics of early biofilm was evaluated by Biofilm Ring test (BRT) (BioFilm Control, Saint Beauzire, France) as described [59]. This assay is based on the immobilization of magnetic beads embedded by bacterial aggregates with enough strength to overcome the magnetic attraction forces applied on them. Thus, in the absence of sessile cells, all the beads are gathered by a magnet centered under the well, giving an easily detectable red spot [59]. Three experiments with two repeats (2 wells per slide) were performed per strain and per incubation time. Experimental details are given in the supplementary materials (Supplementary Protocol S1).

### Quantification of PIA/PNAG production

The PIA/PNAG polysaccharide of biofilm was quantified by the immuno-slot-blotting method with rabbit anti-PIA/PNAG antiserum as described by Cuçarella *et al.* [60].

### Quantification and visualization of the capsular polysaccharides

Capsular polysaccharides type 5 (CP5) or 8 (CP8) were quantified by the immuno-slot-blotting method with rabbit anti-CP5 or anti-CP8 antisera, essentially as described by Luong *et al.* [24]. CP8 production was determined by indirect immunofluorescence. After culture for 8 h in BHI medium at 37°C, bacteria were spotted in diagnostic slides. The slide was incubated with rabbit anti-CP8 antiserum for 1 h at 37°C in humid chamber. After 2 washes, the slide was incubated with secondary antibody, AlexaFluor 647 goat anti-rabbit immunoglobulin G (Invitrogen, Paisley, UK). Total bacteria were labeled with a 1∶1 mixture of vancomycin and vancomycin-Bodipy FL (VBFL) fluorochrome (Invitrogen) at a concentration of 0.8 µg/ml for 15 min. Images were analyzed with confocal microscopy Leica SP5 X (Leica, Solms, Germany) and treated with Leica software: LAS AF Lite (Leica Application Suite 2.6.0).

CP5 or CP8 were quantified by the immuno-slot-blotting method with rabbit anti-CP5 or anti-CP8 antisera, essentially as described by Luong *et al.* [24]. Experimental details are given in the supplementary material (Supplementary Protocol S1).

### Model of catheter infection in mice

The catheter infection model was performed essentially as described by [61]. C57Bl/6 female mice (11–14 weeks old) were purchased from Charles River Laboratories (Charles River, Wilmington, USA) and maintained in pathogen-free conditions at the “Plateau de Biologie Expérimentale de la Souris” (PBES, Ecole Normale Supérieure de Lyon, Lyon, France). This study and procedure were approved by the “Comité Rhône-Alpes d'Ethique pour l'Expérimentation Animale” (CECCAPP, Lyon, France).

Mice were anesthetized with 2–3% of isofluran. After depilating and disinfecting the back of the mouse, a sterile catheter was implanted subcutaneously on the back of each mouse. Twenty-five microliters of PBS containing approximately 4×10^4^ bacteria (Becker or Becker-Δ*rsa*A) were then injected into the catheter. The wound was sewed by a clip. Each day, mice were weighed and the size of the swelling was measured with an electronic caliper. On days 3, 6 and 10, mice were euthanized by cervical dislocation, the catheter and surrounding tissues were carefully removed and transferred separately in sterile tubes for bacterial quantification by plate counting as described. Experimental details are given in the supplementary materials (Supplementary Protocol S1).

### Bacteremia model

CD-1 female mice (8 to 10 weeks old) were obtained from Charles River Laboratories and maintained in pathogen-free conditions at the PBES. This study and procedure were approved by the CECCAPP. Groups of six mice were inoculated intraperitoneally with 5×10^6^ CFU/mouse in 0.5 ml of PBS. After 1 and 3 h, mice were sacrificed and 4 ml of PBS were intraperitoneally injected to recover the surviving bacteria. Blood samples were also collected in heparinized tubes from intracardiac puncture. Spleen and kidneys were collected after 24 and 48 h. After addition of 1 ml of NaCl 0,9%, organs were crushed with a homogenizer T18 basic. Bacterial quantification of all samples was performed by plate counting.

### Statistical analysis

All the data were expressed as mean ± SD. The statistical analyses were performed using GraphPad Prism 6 software. A one-way analysis of variance was realized to test the results obtained in mature biofilm quantification. Other data were analyzed using Mann-Whitney test. A value of p<0.05, <0.005 or <0.0005 was considered to be statistically significant (*), highly significant (**), or extremely significant (***), respectively.

## Supporting Information

Figure S1 **Effect of RsaA on the** ***S. aureus*** **proteome.** (A) 2-D fluorescence difference gel electrophoresis (DiGE) performed on HG001 and Δ*rsa*A-HG001 strains. Total protein extracts were prepared from cultures performed at 37°C stopped at the stationary phase of growth, and labeled with different cyanines. Yellow spot, for unchanged proteins; green spot, protein synthesis activated in strain expressing RsaA; red spot, protein synthesis repressed in strain expressing RsaA. (B) Interpretation of the LC-MS-MS data. A Spectral Counting (SpC) strategy was carried out using the Mascot identification results and Proteinscape 3.1 package. The total number of proteins and spectra are given for each assay. To normalize data, a correction factor was applied for each condition according to the average spectra number for all samples. Three independent experiments were done. In the second table, the normalized spectral counts SpC for MgrA and for the ribosomal protein L21 (RL21) are given and the data have been normalized to the wild-type (WT) strain. STD: A statistical analysis was performed to provide the mean and the standard deviations, which are represented on the histogram. The data were done on crude extracts prepared from late-exponential growth of the WT (HG001), the Δ*rsa*A mutant strain (Δ*rsa*A), and Δ*rsa*A mutant strain complemented with a plasmid expressing RsaA under its own promoter (Δ*rsa*A/pl. *rsa*A).
(EPS)

Figure S2 **Secondary structures of RsaA and of** ***mgr*** **A mRNA.** (A) RsaA secondary structure. The conserved nucleotides are shown in red. The two regions of interaction (sites 1 and 2) are enboxed. (B) Structure of the leader region of *mgr*A mRNA expressed from the P2 promoter. The Shine and Dalgarno sequence (SD) and the AUG codon are in red. (C) RNase III cleavage assays on *mgr*A mRNA. Lanes 1–2: incubation controls performed on *mgr*A mRNA in the absence (lane 1) or in the presence of 250 nM RsaA (lane 2); lanes 3–5: RNase III (0.65 µM) cleavage assays on *mgr*A in the presence of increasing concentrations of RsaA (25 nM, lane 4; 50 nM, lane 5; 250 nM, lane 6); lane 7: RNase III (0.65 µM) cleavage assay performed on *mgr*A bound to RsaA (250 nM) in a buffer containing Ca^2+^ instead of Mg^2+^. Lanes 8–12: same legend as for lanes 3–7, respectively except that the concentration of RNase III was 0.35 µM. Lanes U, C, G, A: sequencing ladders.
(EPS)

Figure S3 **Effect on RsaA on ica-dependent biofilm formation.** Dot-blot analysis of PIA/PNAG polysaccharide accumulation in wild-type strains and their corresponding Δ*rsa*A mutant strains and the same strains complemented with a plasmid expressing RsaA. Growth was performed in BHI medium containing 0.25% glucose for 16 h. Standardized bacterial suspensions were treated with proteinase K, and 1/5 serial dilutions were spotted on nitrocellulose membrane. PIA/PNAG production was detected with an anti-PIA/PNAG antibody.
(EPS)

Figure S4 **RsaA affects opsonophagocytosis.** Mortality of bacteria after opsonophogocytosis assay. Each *S. aureus* strain was mixed with the same quantity of human serum and PMNs for 30 min at 37°C. Sample dilutions were made in sterile deionized water, and bacterial killing was estimated by plating the diluted samples in duplicate on GP. The cell killing was defined as the reduction in CFU/ml after 30 min compared with that at time zero. The graphic represents the % of mortality for each strain of 3 to 5 individual experiments. Three different *S. aureus* backgrounds (HG001, Newman, Becker) were analyzed, as well as their corresponding Δ*rsa*A mutant strains and the same mutant strains complemented with a plasmid expressing RsaA.
(TIFF)

Figure S5 **The impact of RsaA on virulence in** ***in vivo*** **bacteremia model.** An *in vivo* bacteremia model has been used to mimic an acute infection with Becker and Newman background. (A, C, D) 5×10^6^ bacteria in 500 µl were injected into peritoneal cavity. After 1 h (C) and 3 h (A, D), viable bacteria into peritoneal cavity were removed, enumerated by serial dilutions and plate counting on agar plates. (B, E, F) Blood samples were also collected in heparinated from intra-cardiac puncture and bacteria were enumerated by serial dilutions and plate counting on agar plates. (G, H) After 24 and 48 h, spleen was collected in the two strain backgrounds (Becker and Newman) and their corresponding Δ*rsa*A mutant strains, and bacteria were enumerated. The results were expressed in number of CFU/ml. Each point designates the number of bacteria for each mouse and the black line represents the mean of each tested condition. Statistical differences * (p<0.05), ** (p<0.005) and *** (p<0.0005) between each groups were obtained by using Mann Whitney test.
(TIFF)

Figure S6 **Expression of RsaA in reference strains and clinical isolates.** The expression of RsaA was quantified and normalized to the level of *gyr*B mRNA expression after *in vitro* culture to the mid- or late-exponential phase for 16 clinical isolates (A) and 6 reference strains (B).
(EPS)

Protocol S1 **Supplementary Material and Methods.**
(DOC)

Table S1 ***In silico*** **prediction of base pairings between RsaA and mRNAs.**
(DOCX)

Table S2 **Strains and plasmids.**
(DOCX)

Table S3 **Primers used in this study.**
(DOCX)
