# Supplementary figures and images for: A Non-Coding RNA Promotes Bacterial Persistence and Decreases Virulence by Regulating a Regulator in Staphylococcus aureus
Source: PLoS Pathog. 2014 Mar 20;10(3):e1003979. doi: 10.1371/journal.ppat.1003979 (PMC3961350; doi:10.1371/journal.ppat.1003979)

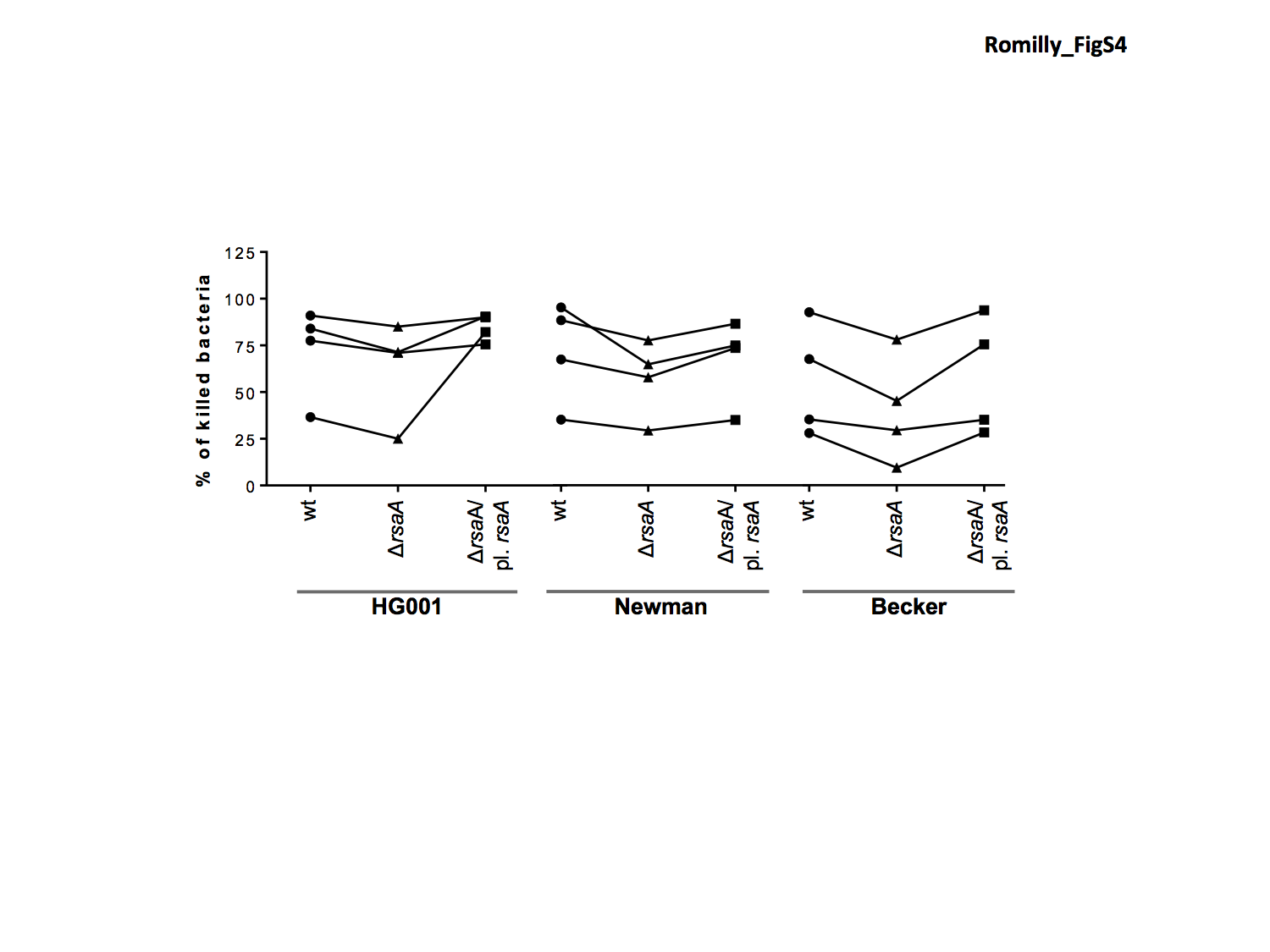

Supplement: Figure S4 — RsaA affects opsonophagocytosis. Mortality of bacteria after opsonophogocytosis assay. Each S. aureus strain was mixed with the same quantity of human serum and PMNs for 30 min at 37°C. Sample dilutions were made in sterile deionized water, and bacterial killing was estimated by plating the diluted samples in duplicate on GP. The cell killing was defined as the reduction in CFU/ml after 30 min compared with that at time zero. The graphic represents the % of mortality for each strain of 3 to 5 individual experiments. Three different S. aureus backgrounds (HG001, Newman, Becker) were analyzed, as well as their corresponding ΔrsaA mutant strains and the same mutant strains complemented with a plasmid expressing RsaA. (TIFF) [file ppat.1003979.s004.tiff]

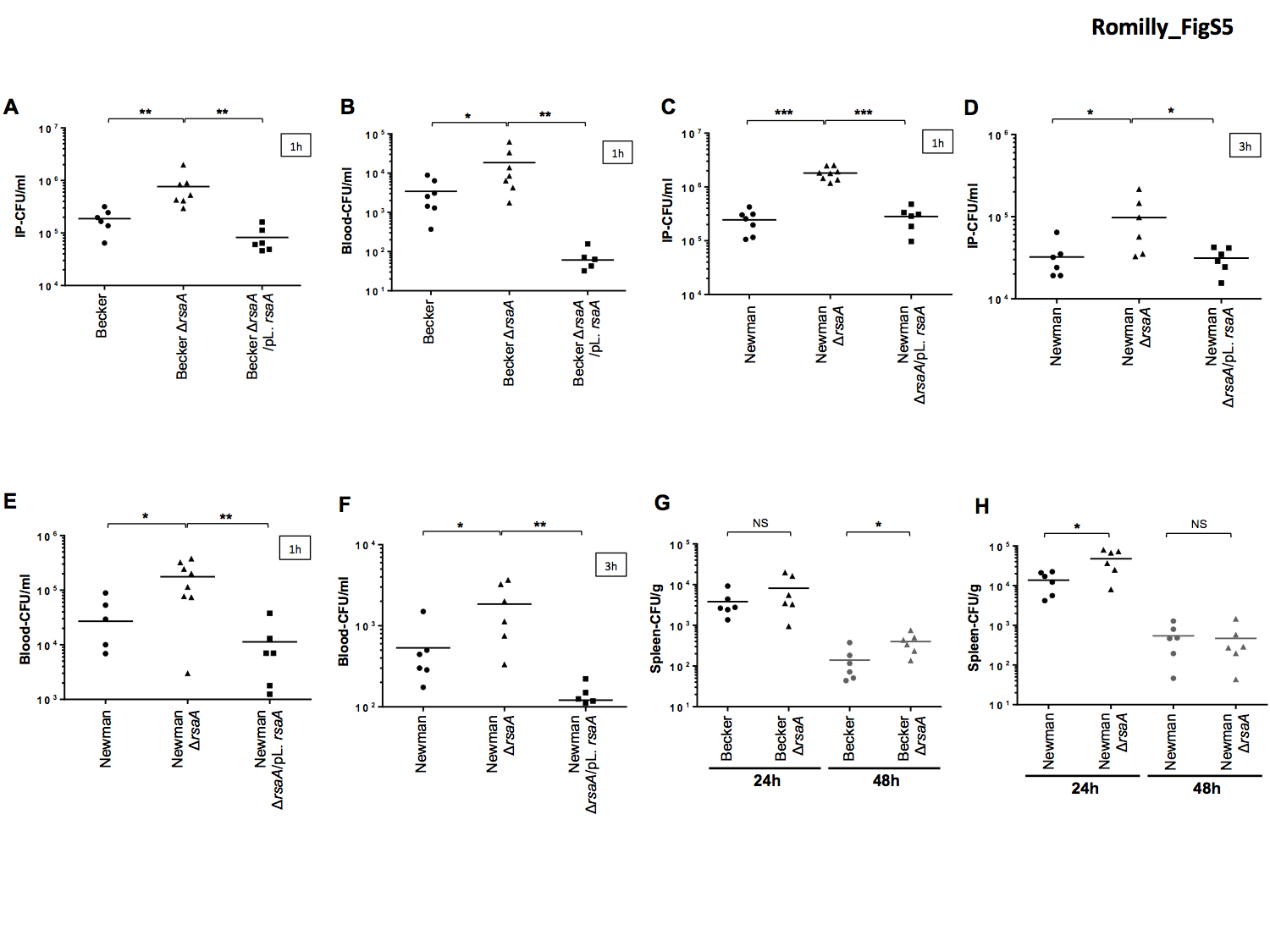

Supplement: Figure S5 — The impact of RsaA on virulence in in vivo bacteremia model. An in vivo bacteremia model has been used to mimic an acute infection with Becker and Newman background. (A, C, D) 5×10^6^ bacteria in 500 µl were injected into peritoneal cavity. After 1 h (C) and 3 h (A, D), viable bacteria into peritoneal cavity were removed, enumerated by serial dilutions and plate counting on agar plates. (B, E, F) Blood samples were also collected in heparinated from intra-cardiac puncture and bacteria were enumerated by serial dilutions and plate counting on agar plates. (G, H) After 24 and 48 h, spleen was collected in the two strain backgrounds (Becker and Newman) and their corresponding ΔrsaA mutant strains, and bacteria were enumerated. The results were expressed in number of CFU/ml. Each point designates the number of bacteria for each mouse and the black line represents the mean of each tested condition. Statistical differences * (p<0.05), ** (p<0.005) and *** (p<0.0005) between each groups were obtained by using Mann Whitney test. (TIFF) [file ppat.1003979.s005.tiff]
