# Supplementary material for: A Non-Coding RNA Promotes Bacterial Persistence and Decreases Virulence by Regulating a Regulator in Staphylococcus aureus
Source: PLoS Pathog. 2014 Mar 20;10(3):e1003979. doi: 10.1371/journal.ppat.1003979 (PMC3961350; doi:10.1371/journal.ppat.1003979)
Supplement: Protocol S1 — Supplementary Material and Methods. (DOC) [file ppat.1003979.s007.doc]

**Supplementary Protocol S1**

**Two-dimensional difference gel electrophoresis (DiGE).**

Strains were grown to post-exponential phase by inoculating 50 ml of BHI medium with an overnight culture (1:100), at 37°C for 7h. The supernatants (1.2 ml) were precipitated by cold acetone, and the pellets washed with cold ethanol. Crude extracts from the pellets were obtained by trizol (Sigma) extraction, followed by acetone precipitation. Protein samples were then pre-labeled with two different fluorescent dyes (Cy3 and Cy5) and were separated by a two-dimensional difference gel electrophoresis (DiGE), which enables quantitative differential display analysis (see Figure S1A). The proteins were dissolved in 7 M urea, 2 M thiourea, 4 % CHAPS, 20 mM Tris-HCl pH 8 and 50 µg were incubated with 400 pmoles of cy2, cy3 or cy5 (Amersham) for 30 min in the dark at 4°C for protein labeling. The reaction was stopped by addition of 20 ml of 10 mM lysine for 10 min. Then, DTT (up to 50 mM) and 10 µl of iodoacetamide 1 M were added to the samples and incubated for 15 min at 37 °C for alkylation. The protein extracts were desalted by a G-25 Sephadex chromatography. Ampholytes (Biorad) and bromophenol blue were added, and the samples were used to re-swell pre-cast pH 3-10 or 4-7 IPG strips (Biorad). Isoelectric focusing was performed using an IEF Cell (Biorad) for a total of 75 kV/h. The IPG strips were buffer-exchanged in 1 M Tris-HCl pH 8.45, 6 M urea, 2 % SDS, 30 % glycerol. For the second dimension, the strips were loaded onto the top of a 12 % polyacrylamide gel- 2 % SDS [1]. After electrophoresis, the gels were scanned for fluorescence detection, and stained with colloidal blue and analyzed using the PQuest software (Biorad). Protein spots excised from the gels were transferred into 96-well microtitration plates. Gel slices were washed with three cycles of incubations in 25 mM ammonium bicarbonate for 15 min and then in 25 mM ammonium bicarbonate containing 50% (v/v) acetonitrile for 15 min. Samples were dehydrated with 100% acetonitrile and reduced with 10 mM DTT for 1h at 56°C. Proteins were alkylated with 55 mM iodoacetamide for 1h in the dark at 20°C. Gel pieces were washed again with the destaining solutions and 20 ng of modified sequencing-grade trypsin (5 ng/µL) in 25 mM ammonium bicarbonate was added on each dehydrated gel piece. After 30 min of rehydratation at 20°C, 30 µL of 25 mM ammonium bicarbonate was added on gel pieces before digestion overnight at 37°C. The resulting peptides were extracted from the gel pieces with incubation of 60% acetonitrile and 5% formic acid. The initial digestion and extraction supernatants were vacuum dried in a SpeedVac. Four different experiments were carried out to provide confident statistical analysis.

**Mass spectrometry analysis and label free quantification by spectral count**

Proteins (5 µg), which were precipitated with acetone, were resuspended in 50 mM ammonium bicarbonate. After a reduction-alkylation step (DTT 5 mM-Iodo 10 mM), proteins were digested overnight with 1/25 (W/W) of trypsin, and only 1.5 µg of proteins was vacuum dried. The dried peptides were resuspended in 15 µL of water containing 0.1% FA (solvent A). The peptide mixtures (500 ng) were analyzed using a NanoLC-2DPlus system (with nanoFlex ChiP module; Eksigent, ABSciex, Concord, Ontario, Canada) coupled to a TripleTOF 5600 mass spectrometer (ABSciex) operating in positive mode. Each sample (5 µg) was loaded on a ChIP C-18 precolumn (300 µm ID x 5 mm ChromXP; Eksigent) at 2 µL/min in solvent A. After 10 min of desalting and concentration in the trap, the pre-column was switched online with the analytical ChIP C-18 analytical column (75 µm ID x 15 cm ChromXP; Eksigent) equilibrated in 95% solvent A and 5% solvent B (0.1% formic acid in acetonitrile). Peptides were eluted by using a 5%-40% gradient of solvent B for 120 min at a flow rate of 300 nL/min. The TripleTOF 5600 was first operated in data-dependant acquisition mode with Analyst software (ABSciex). Survey MS scans were acquired during 250 msec in the 350-1250 m/z range. Up to 20 of the most intense multiply charged ions (2+ to 5+) were selected for CID fragmentation, if they exceeded the 150 counts per second intensity threshold. Ions were fragmented using collision energy spread of 45±15eV spread within a 50 ms accumulation time and an exclusion time of 15s. This so-called “Top20” method, with a constant cycle time of 3.3s, was set in high-sensitivity. Each sample was submitted to a “Top20” discovery analysis.

Data were searched against *S. aureus* database. The complete proteome set from the UniProt database was added to human keratins and trypsin sequences, and the final fasta file was created adding a decoy database. The first algorithm used was Mascot (version 2.2, Matrix Science, London, UK) through the ProteinScape 3.1 package (Bruker). Peptide modifications allowed during the search were: N-acetyl (protein), carbamidomethylation (C) and oxidation (M). Mass tolerances in MS and MS/MS were set to 20 ppm and 0.5 Da, respectively. Two trypsin missed cleavages sites were allowed. Peptide identifications obtained from Mascot were validated with a peptide FDR of 1%. A Spectral Counting strategy was carried on using the Mascot identification results and Proteinscape 3.1 package [2, 3]. To normalize the data, a correction factor was applied for each condition according to the average spectra number for all samples.

**Toeprinting assays**

The preparation of the *S. aureus* 30S subunits, the formation of a simplified translational initiation complex with mRNA, and the extension inhibition conditions were described according to Fechter *et al.* [4]. Standard conditions contained 15 nM *mgr*A mRNA annealed to a 5’ end-labeled oligonucleotide complementary to nts +96 to +118 of *mgr*A mRNA, 250 nM *S. aureus* 30S ribosomal subunits (250 nM), and 25 to 100 nM of RsaA or its variants in 10 µl of buffer containing 20 mM Tris-acetate, pH 7.5, 60 mM NH_4_Cl, 10 mM magnesium acetate, and 3 mM ß-mercaptoethanol. After 10 min at 37°C, the initiator tRNA (1 µM) was added and the reaction was incubated for a further 5 min at 37°C. Reverse transcription was conducted with one unit of AMV reverse transcriptase for 15 min at 37°C. Relative toeprinting (toeprint band over full-length RNA+toeprint) was quantified after scanning of the autoradiography.

**Biofilm Crystal Violet Staining**

The biofilm formation was detected by tissue culture plate method that allowed semi-quantitative measurement of biofilm formation as described by Heilmann *et al.* [5] with some modifications. After 24h of growth in 96-well tissue culture plates, bacterial biofilm formed on the bottom of each well was evaluated by crystal violet staining and spectrophotometric measure of the OD_600nm_. The OD mean value is proportional to the density of biofilm formed and stained by crystal violet.

Isolates were inoculated in BHI and incubated for 18h at 37°C. After standardization to OD_600nm_=1, 1:250 suspension in BHI supplemented with glucose 0,25 %, 200 µl per well was seeded in 96-well flat bottom plates (Becton Dickinson, Franklin Lakes, USA). Three wells were used for each isolate. BHI alone was used as control. The plates were incubated for 24h at 37°C. Then, contents of each well were gently removed and wells were washed softly three times with phosphate buffer saline 1X (PBS) (Invitrogen) to remove the planktonic bacteria. Biofilm was colored with 150 µl crystal violet (0.01 % w/v) (Merck, Fontenay-sous-bois, France) for 30 min and excess stain was rinsed off by washing four times with PBS. Then, plates were air-dried for 30 min. The dye bound to biofilm was resolubilized with 100 µl of 33 % (v/v) acetic acid (VWR, Radnor, USA). Then OD_570nm_ was measured in each well with a micro ELISA Auto Reader, Model 680 (BioRad, Hercules, USA). All tests were carried out three times and average OD value was calculated for each strain and for negative controls.

**Biofilm Ring test**

The kinetics of early biofilm was evaluated by Biofilm Ring test (BRT) (BioFilm Control, Saint Beauzire, France). This assay is based on the immobilization of magnetic beads embedded by bacterial aggregates with strength enough to overcome the magnetic attraction forces applied on them. Thus, in the absence of sessile cells, all the beads are gathered by a magnet centered under the well and designed a red spot easily detectable [4].

Overnight cultures in BHI were adjusted at OD_600nm_ = 1 and diluted 1:250 in BHI. This diluted suspension (or sterile BHI for control wells) were mixed with 1 % of toner solution corresponding to suspension of magnetic beads (diameter of 4 μm). The mixtures were homogenized and 200 µl per well (including in 8-wells strips) was loaded in duplicate. After different time points of 37°C incubation, wells was covered with 100 µl of opaque contrast solution. The strips were put in the reader (Biofilm Reader, Saint Beauzire, France) to get a scanned image (I_0_). Then, the strips were placed on the Magnetic Block Test for 1 min and scanned again (I_1_). After magnet contact, free beads were attracted in the center of the bottom of the wells. Biofilm indices (BFI) ranging from 0 to 20 were calculated for each well with the help of biofilm control software by comparison of beads repartition in image I_0_ and I_1_. A ΔBFI was then calculated as the BFI of each sample – the BFI of the control without bacteria. A low ΔBFI value corresponds to high mobility of beads under magnet action while values higher than 2 correspond to a full immobilization of beads by adherent cells, meaning that the biofilm is completely formed. ΔBFI values between 2 to 7 indicate that biofilm formation is in progress. When the BFI reached the threshold of 7, the biofilm is formed. Three experiments with two repeats (2 wells per slide) were performed per strain and per incubation time.

**Quantification of capsular polysaccharides type 5 or 8 production**

Capsular polysaccharides type 5 (CP5) or 8 (CP8) were quantified by the immuno-slot-blotting method with rabbit anti-CP5 or anti-CP8 antisera, essentially as described by Luong *et al.* [6]. Briefly, overnight broth cultures were standardized at OD_600nm_ = 0.5, pelleted and resuspended in 500 μl of PBS 1X. Each suspension was treated successively with the following enzymes at 37°C: 200 μg of lysostaphine/ml (Sigma Aldrich, St. Louis, USA) for 15 min, 300 U of DNase I/ml (Sigma Aldrich) and 100 μg of proteinase K/ml (Sigma Aldrich) for 15 min. The proteinase K was also inactivated at 75°C for 10 min. Serial dilutions were loaded onto a nitrocellulose membrane using a dot blot apparatus (Bio-Rad). Membranes were incubated with rabbit anti-CP5 or anti-CP8 antisera (Nabi, Rockville, USA) for 1h, washed with PBS 1X, and then incubated with horseradish peroxidase conjugated goat anti-rabbit and developed using the peroxidase color development kit (GE Healthcare, Munich, Germany).

***In vitro* phagocytosis assay**

The opsonophagocytic killing assay with human polymorphonuclear leukocytes (PMNs) was performed essentially as described by Xu *et al.* [6] with the following modifications. Normal human serum (NHS) was collected *via* “Etablissement Français du Sang” (EFS) and pooled from three healthy adult volunteers. The NHS was aliquoted and stored at **-**80°C for not more than 3 months. The opsonophagocytic killing assay was performed in polypropylene tubes containing 1 x 10^6^ PMNs, 1 x 10^6^ CFU *S. aureus*, and serum 5% final concentration in a total volume of 500 μl minimal essential medium (Invitrogen). Control samples contained *S. aureus* and PMNs (without serum), *S. aureus* and serum or *S. aureus* alone. The samples were incubated for 30 min at 37°C. Sample dilutions were made in sterile deionized water, and bacterial killing was estimated by plating the diluted samples in duplicate on GP. The percent killing was defined as the reduction in CFU/ml after 30 min compared with that at time zero.

**Model of catheter infection in mice**

Female C57Bl/6 mice (11–14 weeks old) were purchased from Charles River Laboratories (Charles River, Wilmington, USA) and maintained in pathogen-free conditions at the “Plateau de Biologie Expérimentale de la Souris” (PBES, Ecole Normale Supérieure de Lyon, Lyon, France). This study and procedure were approved by the “Comité Rhône-Alpes d'Ethique pour l'Expérimentation Animale” (CECCAPP, Lyon, France).

Mice were anesthetized with 2-3% of isofluran. After shaving and disinfecting the back of the mouse, a sterile catheter was implanted subcutaneously on the back of each mouse. Twenty five microliters of PBS containing approximately 4 x 10^6^ bacteria (Newman or LUG1680) were then injected into the catheter. The wound was closed by a clip. Each day, mice were weighed and the size of the abscess was measured with an electronic caliper. On days 3, 6 and 10, mice were euthanized by cervical dislocation, the catheter and surrounding tissues were carefully removed and transferred separately in sterile tubes.

For catheter treatment, we added 1 ml of working solution (9/10 NaCl 0,9% + 1/10 EDTA 0,9%) in each tube containing an infected catheter. The tubes were vortexed during 45 sec, sonicated 3 min at 120 W and vortexed again 45 sec to separate the bacteria from each other and from the catheter. Serial dilutions of the samples were spread on GP agar plates. Colonies were counted after 24h incubation at 37°C.

After addition of 1 ml of NaCl 0,9%, surrounding tissues were crushed with a homogenizer T18 basic (IKA, Staufen, Germany). Serial dilutions of the samples were spread on GP agar plates. Colonies were counted after 24h incubation at 37°C.

**References**
