# Supplementary material for: A Non-Coding RNA Promotes Bacterial Persistence and Decreases Virulence by Regulating a Regulator in Staphylococcus aureus
Source: PLoS Pathog. 2014 Mar 20;10(3):e1003979. doi: 10.1371/journal.ppat.1003979 (PMC3961350; doi:10.1371/journal.ppat.1003979)
Supplement: Table S1 — In silico prediction of base pairings between RsaA and mRNAs. (DOCX) [file ppat.1003979.s008.docx]

| **TargetRNA1 data** | | |
| --- | --- | --- |
| SAO641  MgrA | Score: -87  Pvalue: 0.00010614 | sRNA 65 UAUGUAGUGAU-AGUAGUUAAAGUUC**UCC**-**C**AAGGAAGACUACUCG 108  \|\|\|\|\| \|\|\|\| \|\| :\|\|\|\|\|\|\|\|\|\|\|\| \| \|\|: \| \|\|\|\| :\|\|  mRNA 16 AUACA--ACUAGUCUGUAAUUUCAAGA**GGAGG**UCUGUAUGAUAGGC -28 |
| SA1990 hypothetical protein | Score: -86  Pvalue: 0.000126456 | sRNA 9 AUUACAAAAAUUGUAUAGAGUAGCGACUGUAUAAUUUCUAUUGAGGUUAACGUUUAUAU 67  \|\|\|\|\|\|\|\|\|\|\|\| \| \| \| :\| \|\|\| \| \|\|\|:\|:\|:\|\|\| \| \|\| \| \|\|\|\|\|\|\|  mRNA 20 UAAUGUUUUUAAAAGA----AGUG-UGAAA-AUU**GAGG**GUAAGUACA---GAAAAUAUA -30 |
| SA1956  truncated-SA | Score: -80  Pvalue: 0.000361639 | sRNA 63 UAUAUGUAGUGAUAGUAGUUAAAGU 87  \|\|\|\| \|:\|:\|\|\|\|\|\|\|\|\|\|\|\|\|\|  mRNA) 13 AUAUUAUUUAUUAUCAUCAAUUUCA -12 |
| set11  superantigen-like protein7 | Score: -79  Pvalue: 0.000430849 | sRNA 54 GUUAACGUUUAUAUGUAGUGAUAGUAGUUAAAGUUC**UCCC**AA 95  \|:\|\|\|\|\|\|\|\| \|\| \| \| \|\|\|\|\|\| :\|\| \|\|\|\|\|\|\|\|\|  mRNA 20 CGAUUGCAAAAAUUAA--AGUAUCAU-GAU---AA**GAGG**GUU -16 |
| SA0266 hypothetical protein | Score: -71  Pvalue: 0.00174806 | sRNA 33 GACUGUAUAAUUUCUAUUGA-GGUUAACGUUUAUAUGUAGUGAUAGUAGUUA 83  \|\| \|:\| \|\|\|\|\|\|\|\|\|\|:: :\|\|\|\| \|\|:\|: \| \|::\| \|\|\| \|:\|\|:\|:\|  mRNA 19 CUAAUA-AUUAAAGAUAGUAGUCAAU-GC**GAGGA**AAUGU-ACUUUUAUUAGU -30 |
| ppnK  inorganic polyphosphate/ATP-NAD kinase | Score: -68  Pvalue: 0.00295453 | sRNA 15 AAAAUUGUAUAGAGUAGCGACUGUAUAAUUUCUAUUGAGGUUAA-CGUUUAUAU 67  \|\|\|\|\|\|\|\|\|\|\|: \|:\| \|\| \| \|\|\|\|\|: :\|\| \|\|\|\|\| \|:\|\|\| :\|\|  mRNA 17 UUUUAACAUAUUGCGUA-CUAA-AUAUUGU**GGAG**-----CAAUUAGUAAAAGUA -30 |
| SA0634 hypothetical protein | Score: -66  Pvalue: 0.00419129 | sRNA 10 UUACAA-AAAUUGU--AUAGAGUAGCGACUGUAUAAUUUCU 47  \|:\|\|\|\| \|\|\|\|::\| \|\| \|\| :\| \| \|\|:\|\|\|\|\|\|\|\|\|\|  mRNA 14 AGUGUUAUUUAGUAGCUAACU-GU-**GAGGA**UAUAUUAAAGA -25 |
| mqo2 malate:quinone oxidoreductase | Score: -66  Pvalue: 0.00419129 | sRNA 3 UUAACCAUUACAAAAAUUGUA 23  \|\| \|\|\| :\| \|\|\|\|\|\|\|\|\|\|\|  mRNA -5 AAGUGGA**GAGG**UUUUUAACAU -25 |
| fruB  fructose 1-phosphate kinase | Score: -64  Pvalue: 0.00594423 | sRNA 34 ACUGUAUAAUUUCUAUUGAGGUUAACGUUUAUAUGUAGUGAUA 76  \|\|\|\|\|\|\|\|\| \|: \|\| \|:\|\| \| :\|: \|\|\|: \|\|\|\|\|\|\|  mRNA 14 UGACAUAUUUAGUAU---UUCA---G**GGAGGA**UAUUUCACUAU -23 |
| mecR1  methicillin resistance protein | Score: -63  Pvalue: 0.00707803 | sRNA 9 AUUACAAAAAUUGUA 23  \|\|:\|\|\|\|\|\|\|\|\|:\|\|  mRNA -16 UAGUGUUUUUAAUAU -30 |

| **intaRNA data** | | |
| --- | --- | --- |
| SAO641  MgrA | **Energy: -17.9 kcal/mol**  Hybridation: -35.8 kcal/mol  Unfolding mRNA: 8.2 kcal/mol  Unfolding RsaA: 9.7 kcal/mol | SA0641 -29 ACGGAUAGUAUGUCUGG**AGGAG**AACUUUAAUGUCUGAUCA--ACAUA 16  \|\| \|\|\|\| \|\|: \|\|\|\|\|\|\|\|\|\|\|\|: \|\|\|\| \|\|\|\|\|  RsaA 109 GGCUCAUCAGA-AGGAA**CCCUC**UUGAAAUUGAUGA-UAGUGAUGUAUA 64 |
| SA1956  truncated-SA | **Energy: -17.8 kcal/mol**  Hybridation: -27.4 kcal/mol  Unfolding mRNA: 1.4 kcal/mol  Unfolding RsaA: 8.2 kcal/mol | SA1956 -27 AUCU CCUCUUAAACUACUUUAACUACUAUUAUUUAUUAUAC 14  \|\|\| \|\|\| \|\|\|\|\|\|\|\|\|\|\|\|\|\|:\|:\| \|\|\|\|  RsaA 102 CAGAAGGAA**CCCU**CU-UGAAAUUGAUGAUAGUGAUGUAUAUU 62 |
| SA1001  Formyl peptide inhibitory protein | **Energy: -13.7 kcal/mol**  Hybridation: -23.5 kcal/mol  Unfolding mRNA: 2.5 kcal/mol  Unfolding RsaA: 7.4 kcal/mol | SA1001 -14 AG**GGAG**AACUU--AUUAUGAAAAAAAAAUAUCACA 18  \|\|\|\|\|\|\|\|\|\| \|:\|\|: \|\|\|\|\|\|\|  RsaA 94 A**CCCU**CUUGAAAUUGAUG----------AUAGUGA 70 |
| SA2353  hypothetical protein | **Energy: -13.5 kcal/mol**  Hybridation: -27.2 kcal/mol  Unfolding mRNA: 5.5 kcal/mol  Unfolding RsaA: 8.2 kcal/mol | SA2353 -30 -GU-GUUUUGAUUAUUG**GGAGG**AUAUUUAAUUAUGAAAAAAUCGUUACAG 19  \|\| \|\|:\|\| \|\|\|\|\|\|\|:\|: \|\|\|\|\|:\|\|: \|\|\|::\|\|\|\|  RsaA 106 UCAUCAGAAGG---AA**CCCU**CUUGAAAAUUGAUG------UAGUGAUGUA 66 |
| SA1833  hypothetical protein | **Energy: -13.3 kcal/mol**  Hybridation: -26.6 kcal/mol  Unfolding mRNA: 5.1 kcal/mol  Unfolding RsaA: 8.2 kcal/mol | SA1833 -30 GUAAGUCAAAAACGA**AGGAGG**AUUUUAAUUAUG---ACUAUUUU--AGCG 15  \|\| \|\|\|\| \| \|\|\|\|:\|:\|\|\|\|\|:\|\|: \|\|\|\|: \|:\|  RsaA 105 CA-UCAGAAG--GAA**CCCUC**UUGAAAUUGAUGAUAGUGAUGUAUAUUUGC 59 |
| SA0424  hypothetical protein | **Energy: -13.1 kcal/mol**  Hybridation: -22.5 kcal/mol  Unfolding mRNA: 1.6 kcal/mol  Unfolding RsaA: 7.9 kcal/mol | SA0424 -10 ACU**AGGGGG**AAUU---AUUAUUAUGGCA 15  \|\| \|\|\|:\|\|\|:\| \|:\|\|:\|\|\| :\|  RsaA 97 GGAA**CCCU**CUUGAAAUUGAUGAUAGUG 70 |
| SA2485 hypothetical protein | **Energy: -13.0 kcal/mol**  Hybridation: -24.8 kcal/mol  Unfolding mRNA: 3.6 kcal/mol  Unfolding RsaA: 8.2 kcal/mol | SA2485 -28 UGUUUUAUUUAUGGG**GGAGG**---AAUUAAUAAUGACUACAA--AAACA 15  \|\|:\|\| \|\|\|\|:\|\|: \|\|:\|\| \|\| \|\|\|\|\|\| \|\|\|\|  RsaA 103 UCAGAAGGA--A**CCCU**CUUGAAAUUGAUGA-UAGUGAUGUAUAUUUGC 59 |
| SA2332  hypothetical protein | **Energy: -12.9 kcal/mol**  Hybridation: -16.4 kcal/mol  Unfolding mRNA: 0.4 kcal/mol  Unfolding RsaA: 3.0 kcal/mol | SA2332 -15 ACUUG**GGAG**AU -5  \|\|\|\|\|\|\|\|\|  RsaA 97 GGAA**CCCU**CUU 87 |
| SA2271  hypothetical protein | **Energy: -12.9 kcal/mol**  Hybridation: -28.5 kcal/mol  Unfolding mRNA: 5.9 kcal/mol  Unfolding RsaA: 9.7 kcal/mol | SA2271 -26 GCGAGCCAGAUUGA--UGGGAUGAUG--UAAUGU---UUACUACGUAUAAAA 19  \|\|\|\| \|\| :\| \|\|\|\|\| \|\| \|\|\|: \|:\|\|\|\|\|:\|\|\|\|\|\|  RsaA 109 GGCUCA-UCAGAAGGAA**CCCU**-CUUGAAAUUGAUGAUAGUGAUGUAUAUUUG 60 |
| ribH  6.7-dimethyl-8-ribityllumazine synthase | **Energy: -12.8 kcal/mol**  Hybridation: -25.2 kcal/mol  Unfolding mRNA: 4.3 kcal/mol  Unfolding RsaA: 8.2 kcal/mol | ribH -28 GGGUCAUUUAAUAU**AGGAGG**ACUUUAAC-AUGAAUUUUGAAGGUAAAUU 20  :\|\|\| \|\|: \|\|\|\|:\|\|\|\|\|\|\|\| \|: :\|: :\|\|\|\|:  RsaA 104 AUCAG-AAGGAA**C**--**CCU**CUUGAAAUUGAUGAUAGUGAUGUAUAUUUGC 59 |
| GlyS  Glycyl-tRNA synthetase | **Energy: -12.8 kcal/mol**  Hybridation: -27.2 kcal/mol  Unfolding mRNA: 5.7 kcal/mol  Unfolding RsaA: 8.7 kcal/mol | glyS -30 GGGC-GUUUUCAUGUAUGA**GGAG**AGG—-UAAUUAUGG-CAAAAGAUAUGGAUA 19  \|:\| \|\|:\|\|\| \|\| \|\|\|\|\|: \|\|\|:\|\|: \|\| \| \|\|\|\|::\|:  RsaA 107 CUCAUCAGAAGGA---A**C**-**CCU**CUUGAAAUUGAUGAUAGUGAUGUAUAUUUGC 59 |

| **Mfold data** | | |
| --- | --- | --- |
| YabJ-SopVG | **Energy: -15.3 kcal/mol**  **Energy: -21.9 kcal/mol** | YabJ -30 CUAAGUUUUCAUAAA**AGGAG**UUUUAGUAUUAUGAAAAUCAUUAACACAACAAGAUUACCG +20  \|\|\|:\|\|\| \|\|\|\| \|\| \|:\|\|: \|\|\|\|:\| \|\| \| \|\|\|  RsaA 60 UCAUCAGAAGGAA**C**—-**CCU**CUUGAAAUUGAUGA---UAGUGAU-GUAUA-UUUG 100  SpoVG -20 ACUAU**AGGGGG**GC-----------UCACUACAUG-AAA-GU-GACA +12  \|\| \|\|:\|::\| \|\|\|\|\|\|\|\|\|: \|\|\| \|\| :\|\|  RsaA 97 GGA-A**CCCU**CUUGAAAUUGAUGAUAGUGAUGUAUAUUUGCAAUUGG 53 |

**Table S1. *In silico* prediction of base pairings between RsaA and mRNAs.**
