# Supplementary material for: A Non-Coding RNA Promotes Bacterial Persistence and Decreases Virulence by Regulating a Regulator in Staphylococcus aureus
Source: PLoS Pathog. 2014 Mar 20;10(3):e1003979. doi: 10.1371/journal.ppat.1003979 (PMC3961350; doi:10.1371/journal.ppat.1003979)
Supplement: Table S2 — Strains and plasmids. (DOCX) [file ppat.1003979.s009.docx]

|  | **Table S2. Strains and plasmids.** | |  |
| --- | --- | --- | --- |
|  |  | **Relevant characteristics** | **Reference** |
| ***S. aureus strains*** | |  |  |
|  | 8325-4 | NCTC8325 cured of three prophages | Novick (1963) |
|  | HG001 | Derivative of 8325-4, *rsb*U restored RN1, *agr* positive | Herbert *et al.* (2010) |
|  | RN4220 | Restriction mutant of 8325-4 that accepts foreign DNA | Kreiswirth *et al.* (1983) |
|  | RN6390 | Derivative of 8325-4, *rsb*U deficient, *agr* positive | Peng *et al.* (1988) |
|  | Newman | Clinical isolate, high level of clumping factor (ATCC25904), CP5 strain | Duthie *et al.* (1952) |
|  | Becker | CP8 strain - high level capsule production | Sau *et al.* (1996) |
|  | LAC | CA-MRSA USA300 lineage, CC8, ST8 | Voyich *et al*. (2006) |
|  | SF8300 | CA-MRSA USA300 lineage, CC8, ST8 | Diep *et al*. (2008) |
|  | HT2002-0209 | CA-MRSA European lineage, CC80 , ST80 | Perret *et al.* (2012) |
|  | LUG1450 | RN6390 Δ*rsa*A | This study |
|  | LUG1426 | RN6390/pLUG754 | This study |
|  | LUG1740 | LUG1450/pLUG959 | This study |
|  | LUG 1630 | HG001 Δ*rsa*A | This study |
|  | LUG1644 | HG001/pLUG754 | This study |
|  | LUG1741 | LUG1630/pLUG959 | This study |
|  | LUG1664 | Newman/pLUG754 | This study |
|  | LUG1680 | Newman Δ*rsa*A | This study |
|  | LUG1742 | LUG1680/pLUG959 | This study |
|  | LUG1923 | Newman/pLUG298 | This study |
|  | LUG1924 | HG001/pLUG298 | This study |
|  | LUG1933 | Becker/pLUG298 | This study |
|  | LUG2004 | Becker/pLUG754 | This study |
|  | LUG2010 | Becker Δ*rsa*A | This study |
|  | LUG2032 | LUG2010/pLUG959 | This study |
|  | LUG2009 | Becker Δ*rsa*A Δ*mgr*A | This study |
|  | LUG2031 | LUG2009/pLUG959 | This study |
|  | CYL1040 | Becker Δ*mgr*A | Luong *et al.* (2003) |
| ***S. aureus clinical isolates*** | | |  |
|  | ST 2010 1963 | Suppurative cutaneous infection MSSA CC10 | Song *et al.* (2012) |
|  | ST 2011 0136 | Suppurative cutaneous infection MSSA CC398 | Song *et al.* (2012) |
|  | A880740 | CA-pneumonia MSSA CC121 | Gillet *et al.* (2002) |
|  | ST2011 0171 | Sputum of cystic fibrosis patient CC5 | Song *et al.* (2012) |
|  | ST 2011 0214 | Sputum of cystic fibrosis patient CC398 | Song *et al.* (2012) |
|  | HT2003-0826 | CA-pneumonia MRSA CC152/377 | Gillet *et al.* (2002) |
|  | LY1999-0053 | CA-pneumonia MRSA CC80, ST80 | Gillet *et al.* (2002) |
|  | LY1999-0299 | CA-pneumonia MSSA CC121 | Gillet *et al.* (2002) |
|  | LY1999-0310 | CA-pneumonia MSSA CC15 | Gillet *et al.* (2002) |
|  | LY1999-0604 | CA-pneumonia MSSA CC30 | Gillet *et al.* (2002) |
|  | ST2008-0594 | CA-endocarditis CC30 | Tristan *et al.* (2012) |
|  | ST2008-0783 | CA-endocarditis CC5 | Tristan *et al.* (2012) |
|  | ST2009-0228 | CA-endocarditis CC45 | Tristan *et al.* (2012) |
|  | ST2010-2175 | Nasal colonisation MSSA CC9 | Song *et al.* (2012) |
|  | ST2010-2221 | Nasal colonisation MSSA CC398 | Song *et al.* (2012) |
|  | ST2011-1457 | CA-pneumonia MRSA USA300 CC8 | Sicot *et al.* (2012) |
|  | ST2012-0543 | CA-pneumonia MRSA USA300 CC8 | Sicot *et al.* (2012) |
|  | ST2012-2003 | CA-pneumonia MRSA USA300 CC8 | Sicot *et al.* (2012) |
|  |  |  |  |
| ***Plasmids*** | |  |  |
|  | pMAD | Thermosensitive origine of replication, constitutively expressed *bga*B gene | Arnaud *et al.* (2004) |
|  | pE194 | 3.728 kb *S. aureus* plasmid, inductible MLS resistance (*erm*) | Horinouchi and Weiblum (1982) |
|  | pLUG274 | pE194::EcoRV site in MCS | Benito *et al.* (2000) |
|  | pLUG298 | pLUG274::P3 operon (nts 1819-751) | Huntzinger *et al.* (2005) |
|  | pLUG754 | pMAD::*rsa*A(nts 76-1120)-*kan*A*-rsa*A(nts 1246-2194) | This study |
|  | pLUG959 | pLUG274::P3 promoter (nts 1819-1569)::*rsa*A | This study |
|  | pLUG220 | pTCV-lac delta RBS and start codon | Huntzinger et al. 2005 |
|  | pLUG220 P*rpo*B-P1-*mgr*A | pLUG220 with translational fusion *mgr*A::*lacZ* (short fragment) | This study |
|  | pLUG220 P*rpo*B-P1-S1-*mgr*A | pLUG220 with translational fusion mgrA::*lacZ* (short fragment) with RsaA | This study |
|  | pLUG220 P*rpo*B-P1-S2-*mgr*A | pLUG220 with translational fusion *mgr*A::*lacZ* (mutagenesis of RsaA binding site 2 on *mgr*A mRNA) | This study |
|  | pCN38 | Shuttle vector | Charpentier and al. 2004 |
|  | pCN38-PσB-*rsa*A | pCN38 expressing RsaA under its own promoter | This study |
|  | pCN38-PσB-*rsa*A-S1 | pCN38 expressing RsaA under its own promoter, mutated for mgrA binding site 1 | This study |
|  | pCN38-PσB-*rsaA*-S2 | pCN38 expressing RsaA under its own promoter, mutated for mgrA binding site 2 | This study |
|  | pUC18-*rsa*A | *rsa*A under the control of T7 promoter | This study |
|  | pCN51 | Shuttle vector | Charpentier and al. 2004 |
|  | pCN51-PσB-*rsa*A | pCN51 expressing RsaA under its own promoter | This study |
|  |  |  |  |
|  |  |  |  |
|  |  |  |  |
