# Supplementary material for: A Non-Coding RNA Promotes Bacterial Persistence and Decreases Virulence by Regulating a Regulator in Staphylococcus aureus
Source: PLoS Pathog. 2014 Mar 20;10(3):e1003979. doi: 10.1371/journal.ppat.1003979 (PMC3961350; doi:10.1371/journal.ppat.1003979)
Supplement: Table S3 — Primers used in this study. (DOCX) [file ppat.1003979.s010.docx]

**Table S3. Primers used in this study**

| **Primer** | **strand** | **Sequence** | **Use** |
| --- | --- | --- | --- |
| kcr3 | + | CGTTTCTGCG GACTGG | Inactivation control |
| kcr4 | - | CTATCGCCTT CTTGACG | Inactivation control |
| rsaA-24 | + | GGTGCATCAA AAATGACG | Inactivation control |
| rsaA-76 | + | AGCCCGGGAT CCAAAACATA GTC | Plasmid construction |
| rsaA-1120 | - | TGGATATCTA ATATTATTTT AACCTATTTG | Plasmid construction |
| rsaA-1246 | + | ACATCGATAT TGATAATACA TTAGC | Plasmid construction |
| rsaA-2194 | - | TTGTCGACAA GGAGCGATAA ACATG | Plasmid construction |
| rsaA-2270 | - | GCCACGTCAC CAATTTG | Inactivation control |
| pRsaA | + | cgcgcatgcttagtagttaaaattctcttg | Plasmid construction |
| pT2 | - | cgcctgcagaataaaaaaattccaagcttatcggt | Plasmid construction |
| T7 P1 mgrA mRNA for | + | AAAGGATCCTAATACGACTCACTATAGGGGCATACTTTAATTTTAGCATGGC | *in vitro* transciption of *mgrA* mRNA  starting from P1 promoter (short 5'UTR) |
| mgrA rev | - | AGTCACAGGA ATTCCTGCTT GATAGGCTAA GAAACC | in vitro transciption of mgrA mRNA  starting from P1 (short 5'UTR) |
| T7 RsaA BamHI for | + | AAAGGATCCT AATACGACTC ACTATAGGTT AACCATTACA AAAATTGTAT AG | PCR fragment introduced in pUC18, linearized by DraI  use for *in vitro* transcription of RsaA |
| RsaA DraI EcoRI | + | TTGAATTCTT TAAAGTACAC TTTGCTCATA GCAAAGTGTA CCC | PCR fragment introduced in pUC18, linearized by DraI  use for *in vitro* transcription of RsaA |
| mgrA toeprint/RT | + | CAAGAAATTG TGGGTATGTT AG | primer use for reverse transcription for toeprinting assays  complementary to nucleotides 105 to 126 of *mgrA* |
| mgrA mut S1 sens | + | CGGATAGTAT GTCTGGAGGT CTTGTTTAAT GTCTGATCAA CATAATTTAA AAG | mutagenesis of RsaA binding site 1 on *mgrA* mRNA  (AGAAC>TCTTG)  used in PCR on pLUG220-P*rpoB*-P2-*mg*rA |
| mgrA mut S1 rev | - | CTTTTAAATTATGTTGATCAGACATTAAACAAGACCTCCAGACATACTATCCG | mutagenesis of RsaA binding site 1 on *mgrA* mRNA  (ACAAC>TCTTG)  used in PCR on pLUG220-P*rpoB*-P2-*mgr*A |
| mgrA mut S2 sens | + | GTACAATGCTCAAAGACAAGTTAATGCGATGTACTCTAACAAAGTTTTTAAGAAGTAC | mutagenesis mgrA of RsaA binding site 2 on mgrA mRNA  (RsaA binding site) (CGCTAC>GCGATG)  used in PCR on pLUG220-PrpoB-P2-*mgr*A |
| mgrA mut S2 rev | - | GTACTTCTTAAAAACTTTGTTAGAGTACATCGATTAACTTGTCTTTGAGCATTGTAC | mutagenesis of RsaA binding site 2 on *mgrA* mRNA  (GCGATG>CGCTAC)  used in PCR on pLUG220-PrpoB-P2-*mgr*A |
| RsaA mut S1 sens | + | CGTTTATATGTGATAGTAGTTAAACAAGACCCAAGGAAGACTACTCGGGTACAC | mutagenesis of *mgrA* mRNA binding site 1 on RsaA  (TCTTG>AGAAC)  used in PCR on pCN37-PσB-RsaA or pUC-T7-RsaA |
| RsaA mut S1 rev | - | GTGTACCCGAGTAGTCTCTTCCTTGGGTCTTGTTTAACTACTATCACTACATATAAACG | mutagenesis of *mgrA* mRNA binding site 1 on RsaA  (TCTTG>AGAAC)  used in PCR on pCN37-PσB-RsaA or pUC-T7-RsaA |
| RsaA mut S2 sens | + | GGTTAACCATTACAAAAATGTATAGACATCGCACTGTATAATTTCTATTGAGGTTAACG | mutagenesis of *mgrA* mRNA binding site 2 on RsaA  (GTTCT>CAAGA),  used in PCR on pCN37-PσB-RsaA or pUC-T7-RsaA |
| RsaA mut S2 rev | - | CGTTAACCTCAATCAATGAAATTATACAGTGCGATGTCTATCAATTTTTGTAATGGTTAACC | mutagenesis of *mgrA* mRNA binding site 2 on RsaA  (GTTCT>CAAGA),  used in PCR on pCN37-PσB-RsaA or pUC-T7-RsaA |
| PURE system mgrA rev | - | TCGTCCTTGTAGTCTTTTCCTTTGTTTCATCAAATGC | *in vitro* transcription of *mgrA* RNA synthesis with  C-terminal FLAG-Tag for PURE system |
| Translation fusion mgrA P1 BglII sens | + | CTTAAAAGATCTCATACTTTAATTTTAGCATGGC | Translational fusion PrpoB-P1 *mgrA* in pLUG 220  (*mgr*A short 5'UTR) |
| Translation fusion mgrA P2 BglII sens | + | CTTAAAAGATCTGTACGTGGTTTTACCACTAC | Translational fusion PrpoB-P2 *mgrA* pLUG 220  (*mgr*A large 5'UTR) |
| Translation fusion mgrA aa39 BamHI@sens | + | CCGGAATTTTTAAAGGATCCTGTTAAGACAAGAAATTGTGG | Translational fusion PrpoB-P1 *mgrA* in pLUG 220  (*mgr*A short 5'UTR) |
| rpo729 for EcoRI | - | AATGAATTCGTAAAGGAAAGTGATGC | Translational fusion PrpoB-P1 *mgrA* in pLUG 220  (mgrA short 5'UTR) |
| rpo1215 rev BamHI | + | TCAGGATTCAAAAATTATGTGATCCGC | Translational fusion PrpoB-P1 *mgrA* in pLUG 220  (mgrA short 5'UTR) |
| vlac1 | + | GTTGAATAACACTTATTCCTATC | Sequencing of pLUG220 |
| vlac4 | - | CCTCTTCGCTATTACGC | Sequencing of pLUG220 |
| mgrA forward | + | AGTCACAGGAATTCCATACTTTAATTTTAGCATGGC | Probe for Northern Blot |
| mgrA rev T7 | - | AAAGGATCCTAATACGACTCACACTATAGGGGCTGCTTGATAGGCTAAGAAACC | Probe for Northern Blot |

Underlined are the enzyme restriction sites.
